# The burden of unintentional drowning: global, regional and national estimates of mortality from the Global Burden of Disease 2017 Study

**DOI:** 10.1136/injuryprev-2019-043484

**Published:** 2020-02-20

**Authors:** Richard Charles Franklin, Amy E Peden, Erin B Hamilton, Catherine Bisignano, Chris D Castle, Zachary V Dingels, Simon I Hay, Zichen Liu, Ali H Mokdad, Nicholas L S Roberts, Dillon O Sylte, Theo Vos, Gdiom Gebreheat Abady, Akine Eshete Abosetugn, Rushdia Ahmed, Fares Alahdab, Catalina Liliana Andrei, Carl Abelardo T Antonio, Jalal Arabloo, Aseb Arba Kinfe Arba, Ashish D Badiye, Shankar M Bakkannavar, Maciej Banach, Palash Chandra Banik, Amrit Banstola, Suzanne Lyn Barker-Collo, Akbar Barzegar, Mohsen Bayati, Pankaj Bhardwaj, Soumyadeep Bhaumik, Zulfiqar A Bhutta, Ali Bijani, Archith Boloor, Félix Carvalho, Mohiuddin Ahsanul Kabir Chowdhury, Dinh-Toi Chu, Samantha M Colquhoun, Henok Dagne, Baye Dagnew, Lalit Dandona, Rakhi Dandona, Ahmad Daryani, Samath Dhamminda Dharmaratne, Zahra Sadat Dibaji Forooshani, Hoa Thi Do, Tim Robert Driscoll, Arielle Wilder Eagan, Ziad El-Khatib, Eduarda Fernandes, Irina Filip, Florian Fischer, Berhe Gebremichael, Gaurav Gupta, Juanita A Haagsma, Shoaib Hassan, Delia Hendrie, Chi Linh Hoang, Michael K Hole, Ramesh Holla, Sorin Hostiuc, Mowafa Househ, Olayinka Stephen Ilesanmi, Leeberk Raja Inbaraj, Seyed Sina Naghibi Irvani, M Mofizul Islam, Rebecca Q Ivers, Achala Upendra Jayatilleke, Farahnaz Joukar, Rohollah Kalhor, Tanuj Kanchan, Neeti Kapoor, Amir Kasaeian, Maseer Khan, Ejaz Ahmad Khan, Jagdish Khubchandani, Kewal Krishan, G Anil Kumar, Paolo Lauriola, Alan D Lopez, Mohammed Madadin, Marek Majdan, Venkatesh Maled, Navid Manafi, Ali Manafi, Martin McKee, Hagazi Gebre Meles, Ritesh G Menezes, Tuomo J Meretoja, Ted R Miller, Prasanna Mithra, Abdollah Mohammadian-Hafshejani, Reza Mohammadpourhodki, Farnam Mohebi, Mariam Molokhia, Ghulam Mustafa, Ionut Negoi, Cuong Tat Nguyen, Huong Lan Thi Nguyen, Andrew T Olagunju, Tinuke O Olagunju, Jagadish Rao Padubidri, Keyvan Pakshir, Ashish Pathak, Suzanne Polinder, Dimas Ria Angga Pribadi, Navid Rabiee, Amir Radfar, Saleem Muhammad Rana, Jennifer Rickard, Saeed Safari, Payman Salamati, Abdallah M Samy, Abdur Razzaque Sarker, David C Schwebel, Subramanian Senthilkumaran, Faramarz Shaahmadi, Masood Ali Shaikh, Jae Il Shin, Pankaj Kumar Singh, Amin Soheili, Mark A Stokes, Hafiz Ansar Rasul Suleria, Ingan Ukur Tarigan, Mohamad-Hani Temsah, Berhe Etsay Tesfay, Pascual R Valdez, Yousef Veisani, Pengpeng Ye, Naohiro Yonemoto, Chuanhua Yu, Hasan Yusefzadeh, Sojib Bin Zaman, Zhi-Jiang Zhang, Spencer L James

**Affiliations:** 1 College of Public Health, Medical and Veterinary Science, James Cook University, Douglas, Queensland, Australia; 2 Royal Life Saving Society, Sydney, New South Wales, Australia; 3 School of Public Health and Community Medicine, Faculty of Medicine, University of New South Wales, Sydney, New South Wales, Australia; 4 Institute for Health Metrics and Evaluation, University of Washington, Seattle, Washington, USA; 5 Department of Health Metrics Sciences, School of Medicine, University of Washington, Seattle, Washington, USA; 6 College of Medicine and Health Sciences, Department of Nursing, Adigrat University, Adigrat, Ethiopia; 7 Department of Public Health, Debre Berhan University, Debre Berhan, Ethiopia; 8 James P Grant School of Public Health, BRAC University, Dhaka, Bangladesh; 9 Health Systems and Population Studies Division, International Centre for Diarrhoeal Disease Research, Dhaka, Bangladesh; 10 Evidence Based Practice Center, Mayo Clinic Foundation for Medical Education and Research, Rochester, Minnesota, USA; 11 Carol Davila University of Medicine and Pharmacy, Bucharest, Romania; 12 Department of Health Policy and Administration, University of the Philippines Manila, Manila, Philippines; 13 Department of Applied Social Sciences, Hong Kong Polytechnic University, Hong Kong, China; 14 Health Management and Economics Research Center, Iran University of Medical Sciences, Tehran, Iran; 15 Nursing Department, Wolaita Sodo University, Wolaita Sodo, Ethiopia; 16 Department of Forensic Science, Government Institute of Forensic Science, Nagpur, India; 17 Department of Forensic Medicine and Toxicology, Manipal Academy of Higher Education, Manipal, India; 18 Department of Hypertension, Medical University of Lodz, Lodz, Poland; 19 Polish Mothers’ Memorial Hospital Research Institute, Lodz, Poland; 20 Department of Non-Communicable Diseases, Bangladesh University of Health Sciences (BUHS), Dhaka, Bangladesh; 21 Department of Research, Public Health Perspective Nepal, Pokhara-Lekhnath Metropolitan City, Nepal; 22 School of Psychology, University of Auckland, Auckland, New Zealand; 23 Occupational Health Department, Kermanshah University of Medical Sciences, Kermanshah, Iran; 24 Health Human Resources Research Center, Shiraz University of Medical Sciences, Shiraz, Iran; 25 Department of Community Medicine and Family Medicine, All India Institute of Medical Sciences, Jodhpur, India; 26 Department of Community Medicine, Datta Meghe Institute of Medical Sciences, Deemed University, Wardha, India; 27 The George Institute for Global Health, New Delhi, India; 28 Centre for Global Child Health, University of Toronto, Toronto, Ontario, Canada; 29 Centre of Excellence in Women and Child Health, Aga Khan University, Karachi, Pakistan; 30 Social Determinants of Health Research Center, Babol University of Medical Sciences, Babol, Iran; 31 Department of Internal Medicine, Manipal Academy of Higher Education, Mangalore, India; 32 Research Unit on Applied Molecular Biosciences (UCIBIO), University of Porto, Porto, Portugal; 33 Maternal and Child Health Division, International Centre for Diarrhoeal Disease Research, Dhaka, Bangladesh; 34 Department of Epidemiology and Biostatistics, University of South Carolina, Columbia, South Carolina, USA; 35 Faculty of Biology, Hanoi National University of Education, Hanoi, Vietnam; 36 Research School of Population Health, Australian National University, Action, Australian Capital Territory, Australia; 37 Environmental and Occupational Health and Safety, University of Gondar, Gondar, Ethiopia; 38 Department of Human Physiology, University of Gondar, Gondar, Ethiopia; 39 Public Health Foundation of India, Gurugram, India; 40 Toxoplasmosis Research Center, Mazandaran University of Medical Sciences, Sari, Iran; 41 Department of Community Medicine, University of Peradeniya, Peradeniya, Sri Lanka; 42 Tehran University of Medical Sciences, Tehran, Iran; 43 Center of Excellence in Public Health Nutrition, Nguyen Tat Thanh University, Ho Chi Minh City, Vietnam; 44 Sydney School of Public Health, University of Sydney, Sydney, New South Wales, Australia; 45 Department of Global Health and Social Medicine, Harvard University, Boston, Massachusetts, USA; 46 Department of Social Services, Tufts Medical Center, Boston, Massachusetts, USA; 47 Department of Public Health Sciences, Karolinska Institutet, Stockholm, Sweden; 48 World Health Programme, Université du Québec en Abitibi-Témiscamingue, Rouyn-Noranda, Québec, Canada; 49 REQUIMTE/LAQV, University of Porto, Porto, Portugal; 50 Psychiatry Department, Kaiser Permanente, Fontana, California, USA; 51 School of Health Sciences, A.T. Still University, Arizona, Missouri, USA; 52 Department of Population Medicine and Health Services Research, Bielefeld University, Bielefeld, Germany; 53 Public Health Department, Haramaya University, Harar, Ethiopia; 54 Non-Communicable Diseases (NCD), World Health Organization (WHO), New Delhi, India; 55 Department of Public Health, Erasmus University Medical Center, Rotterdam, Netherlands; 56 Centre for International Health and Section for Ethics and Health Economics, University of Bergen, Bergen, Norway; 57 School of Public Health, Curtin University, Perth, Western Australia, Australia; 58 Center of Excellence in Behavioral Medicine, Nguyen Tat Thanh University, Ho Chi Minh City, Vietnam; 59 Department of Pediatrics, Dell Medical School, University of Texas Austin, Austin, Texas, USA; 60 Kasturba Medical College, Manipal Academy of Higher Education, Manipal, India; 61 Department of Legal Medicine and Bioethics, Carol Davila University of Medicine and Pharmacy, Bucharest, Romania; 62 Clinical Legal Medicine Department, National Institute of Legal Medicine Mina Minovici, Bucharest, Romania; 63 Division of Information and Computing Technology, College of Science and Engineering, Hamad Bin Khalifa University, Doha, Qatar; 64 Qatar Foundation for Education, Science, and Community Development, Doha, Qatar; 65 Department of Community Medicine, University of Ibadan, Ibadan, Nigeria; 66 Department of Family Medicine, Bangalore Baptist Hospital, Bangalore, India; 67 Research Institute for Endocrine Sciences, Shahid Beheshti University of Medical Sciences, Tehran, Iran; 68 School of Psychology and Public Health, La Trobe University, Melbourne, Victoria, Australia; 69 School of Public Health and Community Medicine, University of New South Wales, Sydney, New South Wales, Australia; 70 Institute of Medicine, University of Colombo, Colombo, Sri Lanka; 71 Faculty of Graduate Studies, University of Colombo, Colombo, Sri Lanka; 72 Gastrointestinal and Liver Disease Research Center, Guilan University of Medical Sciences, Rasht, Iran; 73 Social Determinants of Health Research Center, Qazvin, Iran; 74 Health Services Management Department, Qazvin University of Medical Sciences, Qazvin, Iran; 75 Department of Forensic Medicine and Toxicology, All India Institute of Medical Sciences, Jodhpur, India; 76 Hematology-Oncology and Stem Cell Transplantation Research Center, Tehran University of Medical Sciences, Tehran, Iran; 77 Pars Advanced and Minimally Invasive Medical Manners Research Center, Iran University of Medical Sciences, Tehran, Iran; 78 Epidemiology Department, Faculty of Public Health and Tropical Medicine, Jazan University, Jazan, Saudi Arabia; 79 Epidemiology and Biostatistics Department, Health Services Academy, Islamabad, Pakistan; 80 Department of Nutrition and Health Science, Ball State University, Muncie, Indiana, USA; 81 Department of Anthropology, Panjab University, Chandigarh, India; 82 Institute of Clinical Physiology, National Research Council, Pisa, Italy; 83 University of Melbourne, Melbourne, Victoria, Australia; 84 Pathology Department, College of Medicine, Imam Abdulrahman Bin Faisal University, Dammam, Saudi Arabia; 85 Department of Public Health, Trnava University, Trnava, Slovakia; 86 Health Education and Research Department, SDM College of Medical Sciences & Hospital, Dharwad, India; 87 Health University, Rajiv Gandhi University of Health Sciences, Bangalore, India; 88 Ophthalmology Department, Iran University of Medical Sciences, Tehran, Iran; 89 Ophthalmology Department, University of Manitoba, Winnipeg, Manitoba, Canada; 90 Plastic Surgery Department, Iran University of Medical Sciences, Tehran, Iran; 91 Department of Health Services Research and Policy, London School of Hygiene & Tropical Medicine, London, UK; 92 Mekelle University, Mekelle, Ethiopia; 93 Forensic Medicine Division, Imam Abdulrahman Bin Faisal University, Dammam, Saudi Arabia; 94 Breast Surgery Unit, Helsinki University Hospital, Helsinki, Finland; 95 University of Helsinki, Helsinki, Finland; 96 Pacific Institute for Research & Evaluation, Calverton, Maryland, USA; 97 Community Medicine, Manipal Academy of Higher Education, Mangalore, India; 98 Department of Epidemiology and Biostatistics, Shahrekord University of Medical Sciences, Shahrekord, Iran; 99 Department of Nursing, Shahroud University of Medical Sciences, Shahroud, Iran; 100 Non-Communicable Diseases Research Center, Tehran University of Medical Sciences, Tehran, Iran; 101 Iran National Institute of Health Research, Tehran University of Medical Sciences, Tehran, Iran; 102 Faculty of Life Sciences and Medicine, King’s College London, London, UK; 103 Department of Pediatric Medicine, Nishtar Medical University, Multan, Pakistan; 104 Department of Pediatrics & Pediatric Pulmonology, Institute of Mother & Child Care, Multan, Pakistan; 105 General Surgery Department, Carol Davila University of Medicine and Pharmacy, Bucharest, Romania; 106 General Surgery Department, Emergency Hospital of Bucharest, Bucharest, Romania; 107 Institute for Global Health Innovations, Duy Tan University, Hanoi, Vietnam; 108 Department of Psychiatry and Behavioural Neurosciences, McMaster University, Hamilton, Ontario, Canada; 109 Department of Psychiatry, University of Lagos, Lagos, Nigeria; 110 Department of Pathology and Molecular Medicine, McMaster University, Hamilton, Ontario, Canada; 111 Department of Forensic Medicine, Kasturba Medical College, Manipal Academy of Higher Education, Manipal, India; 112 Parasitology and Mycology, Shiraz University of Medical Sciences, Shiraz, Iran; 113 Department of Pediatrics, RD Gardi Medical College, Ujjain, India; 114 Health Sciences Department, Muhammadiyah University of Surakarta, Sukoharjo, Indonesia; 115 Department of Chemistry, Sharif University of Technology, Tehran, Iran; 116 College of Medicine, University of Central Florida, Orlando, Florida, USA; 117 College of Graduate Health Sciences, A.T. Still University, Mesa, Arizona, USA; 118 University Institute of Public Health, University of Lahore, Lahore, Pakistan; 119 Public Health Department, University of Health Sciences, Lahore, Pakistan; 120 Surgery Department, University of Minnesota, Minneapolis, Minnesota, USA; 121 Surgery Department, University Teaching Hospital of Kigali, Kigali, Rwanda; 122 Emergency Department, Shahid Beheshti University of Medical Sciences, Tehran, Iran; 123 Sina Trauma and Surgery Research Center, Tehran University of Medical Sciences, Tehran, Iran; 124 Department of Entomology, Ain Shams University, Cairo, Egypt; 125 Health Economics, Bangladesh Institute of Development Studies (BIDS), Dhaka, Bangladesh; 126 Department of Psychology, University of Alabama at Birmingham, Birmingham, Alabama, USA; 127 Emergency Department, Manian Medical Centre, Erode, India; 128 Department of Health Promotion and Education, Alborz University of Medical Sciences, Karaj, Iran; 129 Independent Consultant, Karachi, Pakistan; 130 College of Medicine, Yonsei University, Seodaemun-gu, South Korea; 131 Division of Cardiology, Emory University, Atlanta, Georgia, USA; 132 Department of Forensic Medicine, Kathmandu University, Dhulikhel, Nepal; 133 Medical Surgical Nursing Department, Urmia University of Medical Science, Urmia, Iran; 134 Emergency Nursing Department, Semnan University of Medical Sciences, Semnan, Iran; 135 Department of Psychology, Deakin University, Burwood, Victoria, Australia; 136 Department of Agriculture and Food Systems, University of Melbourne, Melbourne, Victoria, Australia; 137 Center for Health Resource and Services Research and Development, National Institute of Health Research & Development, Jakarta, Indonesia; 138 Department of Pediatrics, King Saud University, Riyadh, Saudi Arabia; 139 College of Medicine, Alfaisal University, Riyadh, Saudi Arabia; 140 Department of Public Health, Adigrat University, Adigrat, Ethiopia; 141 Argentine Society of Medicine, Buenos Aires, Argentina; 142 Velez Sarsfield Hospital, Buenos Aires, Argentina; 143 Psychosocial Injuries Research Center, Ilam University of Medical Sciences, Ilam, Iran; 144 Division of Injury Prevention and Mental Health Improvement, National Center for Chronic and Non-Communicable Disease Control, Chinese Center for Disease Control and Prevention, Beijing, China; 145 Department of Psychopharmacology, National Center of Neurology and Psychiatry, Tokyo, Japan; 146 Department of Epidemiology and Biostatistics, Wuhan University, Wuhan, China; 147 Global Health Institute, Wuhan University, Wuhan, China; 148 Department of Health Economics and Management, Urmia University of Medical Science, Urmia, Iran; 149 Department of Medicine, School of Clinical Sciences at Monash Health, Monash University, Melbourne, Victoria, Australia; 150 Department of Preventive Medicine, Wuhan University, Wuhan, China

**Keywords:** drowning, burden of disease, global

## Abstract

**Background:**

Drowning is a leading cause of injury-related mortality globally. Unintentional drowning (International Classification of Diseases (ICD) 10 codes W65-74 and ICD9 E910) is one of the 30 mutually exclusive and collectively exhaustive causes of injury-related mortality in the Global Burden of Disease (GBD) study. This study’s objective is to describe unintentional drowning using GBD estimates from 1990 to 2017.

**Methods:**

Unintentional drowning from GBD 2017 was estimated for cause-specific mortality and years of life lost (YLLs), age, sex, country, region, Socio-demographic Index (SDI) quintile, and trends from 1990 to 2017. GBD 2017 used standard GBD methods for estimating mortality from drowning.

**Results:**

Globally, unintentional drowning mortality decreased by 44.5% between 1990 and 2017, from 531 956 (uncertainty interval (UI): 484 107 to 572 854) to 295 210 (284 493 to 306 187) deaths. Global age-standardised mortality rates decreased 57.4%, from 9.3 (8.5 to 10.0) in 1990 to 4.0 (3.8 to 4.1) per 100 000 per annum in 2017. Unintentional drowning-associated mortality was generally higher in children, males and in low-SDI to middle-SDI countries. China, India, Pakistan and Bangladesh accounted for 51.2% of all drowning deaths in 2017. Oceania was the region with the highest rate of age-standardised YLLs in 2017, with 45 434 (40 850 to 50 539) YLLs per 100 000 across both sexes.

**Conclusions:**

There has been a decline in global drowning rates. This study shows that the decline was not consistent across countries. The results reinforce the need for continued and improved policy, prevention and research efforts, with a focus on low- and middle-income countries.

## Introduction

Drowning has been identified as a public health priority by the World Health Organization (WHO)^1^ and is defined as the process of experiencing respiratory impairment from submersion/immersion in liquid, with drowning outcomes classified as death, morbidity and no morbidity.^2^ Fatal drowning is a leading cause of unintentional injury-related mortality worldwide, with approximately 300 000 drowning deaths per annum globally.^3 4^ Drowning disproportionately impacts those from low- and middle-income countries (LMICs), males and children.^5 6^ Having accurate and timely data to aid in the allocation of public health resources and the monitoring of interventions is important for continued implementation of drowning prevention programs.^3 7^

The Global Burden of Diseases, Injuries, and Risk Factors (GBD) Study is a comprehensive assessment of health losses associated with risk factors, and from morbidity and mortality. GBD 2017 produced estimates of all-cause mortality, cause-specific mortality, years of life lost (YLLs), incidence, prevalence, years lived with disability (YLDs) and disability-adjusted life years for 292 different causes of mortality and 354 different causes of non-fatal health loss for 195 countries and territories across a 28-year time span from 1990 to 2017^4 8^. Drowning is one of 30 mutually exclusive causes of injury-related mortality in the GBD 2017 study design and is nested within the unintentional injury category of the GBD 2017 cause hierarchy.^9^

Within injury, drowning has the third highest unintentional mortality rate after road injuries and falls. Across all injury types (both intentional and unintentional), drowning has the fourth highest overall mortality rate after road injuries, falls and interpersonal violence.^9^ As such, there is a need for immediate action at global and local levels to address the circumstances leading to drowning to guide preventive efforts.

When considering YLLs, within the unintentional injuries category, drowning has the second highest YLLs after road injuries, demonstrating the significant impact that drowning has, particularly on children.^9^ Conversely, it has among the lowest YLDs due to the high lethality of the injury.^10^ Recent data from the USA shows that for each drowning death there are 2.5 hospitalisations (ratio 1:2.5), whereas for road injuries this ratio is 1:88 and for falls even higher, 1:229.^11^ Thus, reducing drowning deaths will reduce the overall mortality burden but is likely to have limited impact on morbidity of unintentional injuries.

There is limited information on drowning in LMICs. A recent review of published literature on drowning in LMICs between 1984 and 2015 identified 62 studies, with the majority of these from Asia (56%), followed by Africa (26%).^5^ Further work is required to understand the drowning burden in countries in Latin America, Africa, Asia, and the Pacific.

There has been a reduction, over the last decade, in the estimated annual rate of drowning globally, with the latest GBD study showing a reduction of 17.2% from 2007 to 2017.^4^ There is no clear consensus as to why this reduction has occurred. For LMICs, this is likely related to changes in development and urbanisation,^12^ with the greatest reductions occurring in East Asia and southern sub-Saharan Africa.^9^ A recent review of drowning in South Africa found an increase in drowning across the country with variation based on geography,^13^ demonstrating that the drowning burden is not evenly distributed and that choosing data from a region within a country or a country within a region can potentially skew the findings.

This current study focuses on unintentional drowning, as defined by those International Classification of Diseases (ICD-9/10) codes that are primary unintentional drowning codes (E910 and W65-74). This does not include all drowning deaths, meaning intentional (X71), disaster (X38) and transport (V90, V92)-related drowning are not included, with unspecified effects of immersion (T75.1) and undetermined intent (Y21) redistributed across drowning and other categories. It has been estimated that the unintentional drowning ICD codes account for approximately 40%–50% of all drowning mortality in high-income countries.^14 15^ Given some countries have a higher burden of boating-related^16 17^ and disaster-related^18^ drowning mortality, defining unintentional drowning only by ICD codes W65-74 impacts accurate estimates. Similarly, intentional drowning deaths^19^ must be included in overall all-cause estimates of mortality to further enhance accuracy of estimates.

The goal of this study was to report estimates from GBD 2017 for mortality from unintentional drowning by region and compare these to 1990 estimates. While GBD 2017 also reported estimates for non-fatal cases of drowning, the purpose of this study was to focus exclusively on cause-specific mortality from unintentional drowning.

## Methods

The GBD covers all nation-states using a range of data sources to provide a comprehensive picture of global health. An overview of methods used for GBD 2017 is provided in online supplementary appendix 1 and in other GBD literature.^4 8 20–23^ All analytical code used for GBD 2017 is available online (http://www.ghdx.healthdata.org). This study complies with the Guidelines for Accurate and Transparent Health Estimates Reporting recommendations (online supplementary appendix 2). Analyses were completed using Python V.2.7, Stata V.13.1, or R V.3.3. Statistical code used for GBD estimation is publicly available online at healthdata.org. An overview of methods specific to drowning mortality is as follows:

10.1136/injuryprev-2019-043484.supp1 Supplementary data

10.1136/injuryprev-2019-043484.supp2 Supplementary data

### Cause of death data for drowning

GBD 2017 utilised all available cause of death data for every location in the GBD location hierarchy, which includes 195 countries and territories.^4^ The GBD 2017 cause of death database included vital registration (VR), verbal autopsy, police record data and mortuary data, among other data types. These data undergo extensive quality checks and data processing to ensure comparability between different coding systems from different sources that were collected at different times—for example, to allow for both ICD9-coded and ICD10-coded data to be used in a comparable manner. In addition, the GBD cause hierarchy is mutually exclusive and collectively exhaustive, meaning that each death has one and only one underlying cause of death assigned. Cause of death data also undergo a process known as garbage code redistribution, whereby ill-defined cause of death codes are redistributed to underlying causes of death in the GBD 2017 cause hierarchy. Such processes are described in more detail elsewhere.^4^ For GBD 2017, drowning deaths were identified with ICD9 code E910 and ICD10 codes W65-W74.9. In addition, drowning was a cause of death in verbal autopsy survey instruments, which were a source of cause of death data in many locations including India.

### Cause-specific mortality modelling for drowning

Since GBD estimates outcomes for every location, year, age and sex in the GBD 2017 study design, statistical modelling processes are implemented to predict estimates for where data are missing. GBD 2017 utilised standard GBD methods for modelling cause-specific mortality from drowning.^4^ In particular, the study implemented the Cause of Death Ensemble model (CODEm) approach, which is described in more detail elsewhere^4^ but is summarised as follows in terms of five key principles. First, CODEm uses all available data, as described above. Second, data processing steps are conducted to ensure comparability and quality of the dataset, also as described above. Third, CODEm develops a diverse set of plausible models using different regression forms and different sets of covariates. Fourth, CODEm measures the predictive validity of each model and of an ensemble of all models. Finally, the best model is chosen based on out-of-sample predictive validity testing. The CODEm framework is intended to develop the best possible model for each cause given the availability of existing data and relationships with covariates to help inform model estimates where data are absent or sparse.

Table 1 shows the covariates that were utilised for measuring drowning mortality in CODEm, as well as priors for the specified direction of causality (positive meaning that cause-specific mortality is expected to increase with increases in the level of the covariate) and the level of causality expected for the relationship between the covariate and mortality (‘1’ referring to more proximal associations, and ‘3’ referring to more distal associations). These factors are based on common factors described in the literature and expert opinion. Further work exploring these factors and the impact on drowning would add value.

**Table 1:** Covariates used in unintentional drowning CODEm models

Covariate	Level	Direction
Alcohol (litres per capita)	1	+
Coastal population within 10 km (proportion)	1	+
Landlocked nation (binary)	1	–
Log-transformed summary exposure value for drowning	1	+
Rainfall, lowest quintile	1	–
Rainfall, highest quintile	1	+
Elevation under 100 m (proportion	2	+
Education (years per capita)	3	–
Lag-distributed income per capita	3	No prior
Socio-demographic Index	3	–

CODEm, Cause of Death Ensemble model.

Once CODEm models are fit for drowning, the location-specific estimates are scaled to fit within each parent location (eg, all subnational drowning deaths sum to national drowning deaths, and national drowning deaths sum to global drowning deaths). In addition, cause-specific estimates are scaled to fit within the parent cause (eg, the sum of deaths from each injury equals the deaths for the overall injury cause, and the sum of deaths from each individual cause in the cause hierarchy equals the sum of all-cause mortality). For drowning, these processes led to an increase in 0.82% in terms of drowning deaths across all ages for both sexes in 2017. Results are presented followed by a 95% uncertainty interval (UI) range (online supplementary appendix 1).

### Calculating YLLs for drowning

YLLs are defined as the difference between life expectancy and the age at which a death occurs, based on life tables used in GBD 2017 that estimate the remaining life expectancy for each 5-year age group in all populations greater than 5 million in GBD 2017.^22^ YLLs are an important measure of drowning mortality, since drowning more commonly occurs at early ages, which leads to more YLLs than a death occurring at an older age. The age-specific life expectancies used to calculate YLLs are provided in table 2.

**Table 2:** Age-specific life expectancy used for years of life lost calculation

Age	Life expectancy (years)
0	87.9
1	87.0
5	83.0
10	78.1
15	73.1
20	68.1
25	63.2
30	58.2
35	53.3
40	48.4
45	43.5
50	38.7
55	34.0
60	29.3
65	24.7
70	20.3
75	16.1
80	12.2
85	8.8
90	6.1
95	3.9
100	2.2
105	1.6
110	1.4

### Socio-demographic Index

The Socio-demographic Index (SDI) is an index of socio-economic development which is used as a covariate in various GBD processes as well as a metric to demonstrate burden trends for locations at different levels of development. The index is calculated based on lag-distributed income per capita, which is a smoothed gross domestic product per capita series, mean educational attainment in years and total fertility rate under age 25 years. In its formulation, SDI increases as income and education increase and fertility decreases. The derivation of SDI is described in more detail elsewhere.^8^

## Results

Globally, in 1990, there were 531 956 (UI: 484 107 to 572 854) deaths from unintentional drowning, which decreased by 44.5% (UI: 38.9% to 48.3%) to 295 210 (UI: 284 493 to 306 187) deaths from unintentional drowning in 2017. The global age-standardised cause-specific mortality rate for unintentional drowning was 9.3 (UI: 8.5 to 10.0) per 100 000 in 1990, which decreased by 57.4% (UI: 53.3% to 60.1%) to a rate of 4.0 (UI: 3.8 to 4.1) per 100 000 in 2017 (online supplementary table S1).

10.1136/injuryprev-2019-043484.supp3 Supplementary data

Online supplementary table S1 provides all-age drowning-specific deaths and age-standardised cause-specific mortality rates for 1990 and 2017, as well as the percentage change between 1990 and 2017 for every country, region and super-region in the GBD 2017 location hierarchy. Overall, there was a decrease in drowning rates (except in Tonga) and numbers, with the notable exception of Oceania, which had a near doubling (80.1%) of numbers, led by a large increase in Papua New Guinea (93.4%). Online supplementary table S2 provides the same information for YLLs.

10.1136/injuryprev-2019-043484.supp4 Supplementary data

### Age-standardised cause-specific mortality

**Figure 1:**
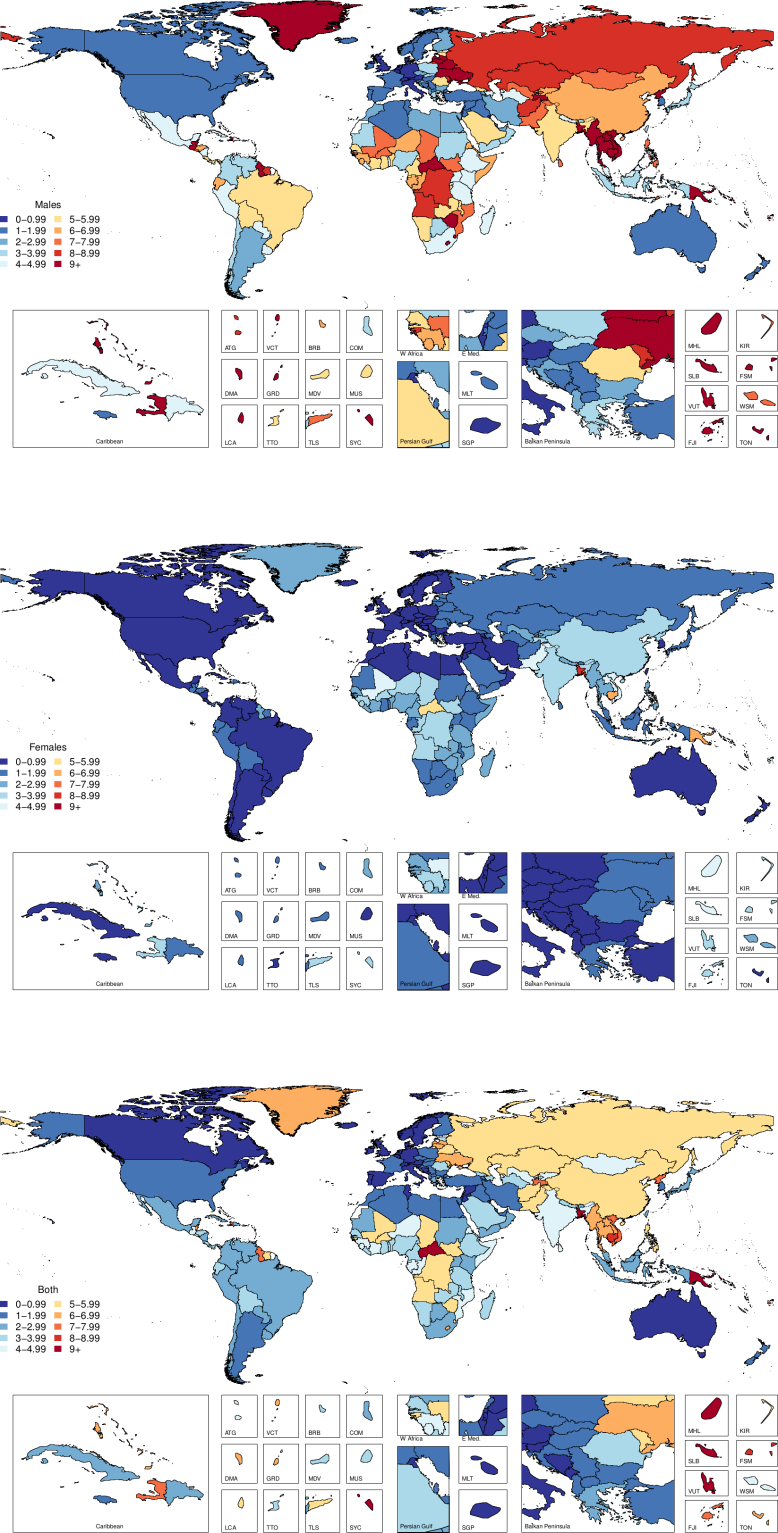
Age-standardised cause-specific mortality rates per 100 000 by country for unintentional drowning in 2017.

**Figure 2:**
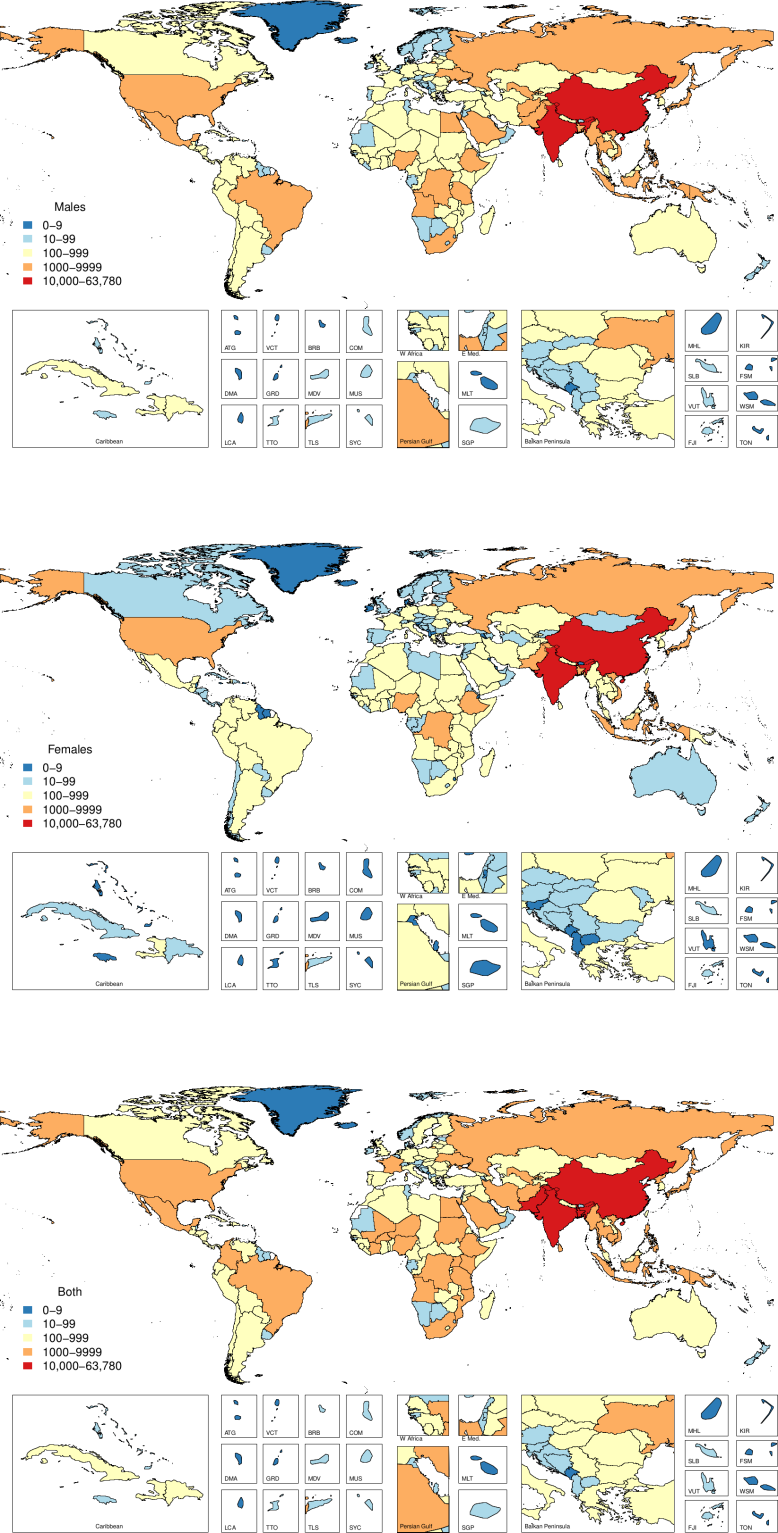
All-age mortality by country for unintentional drowning in 2017.

**Figure 3:**
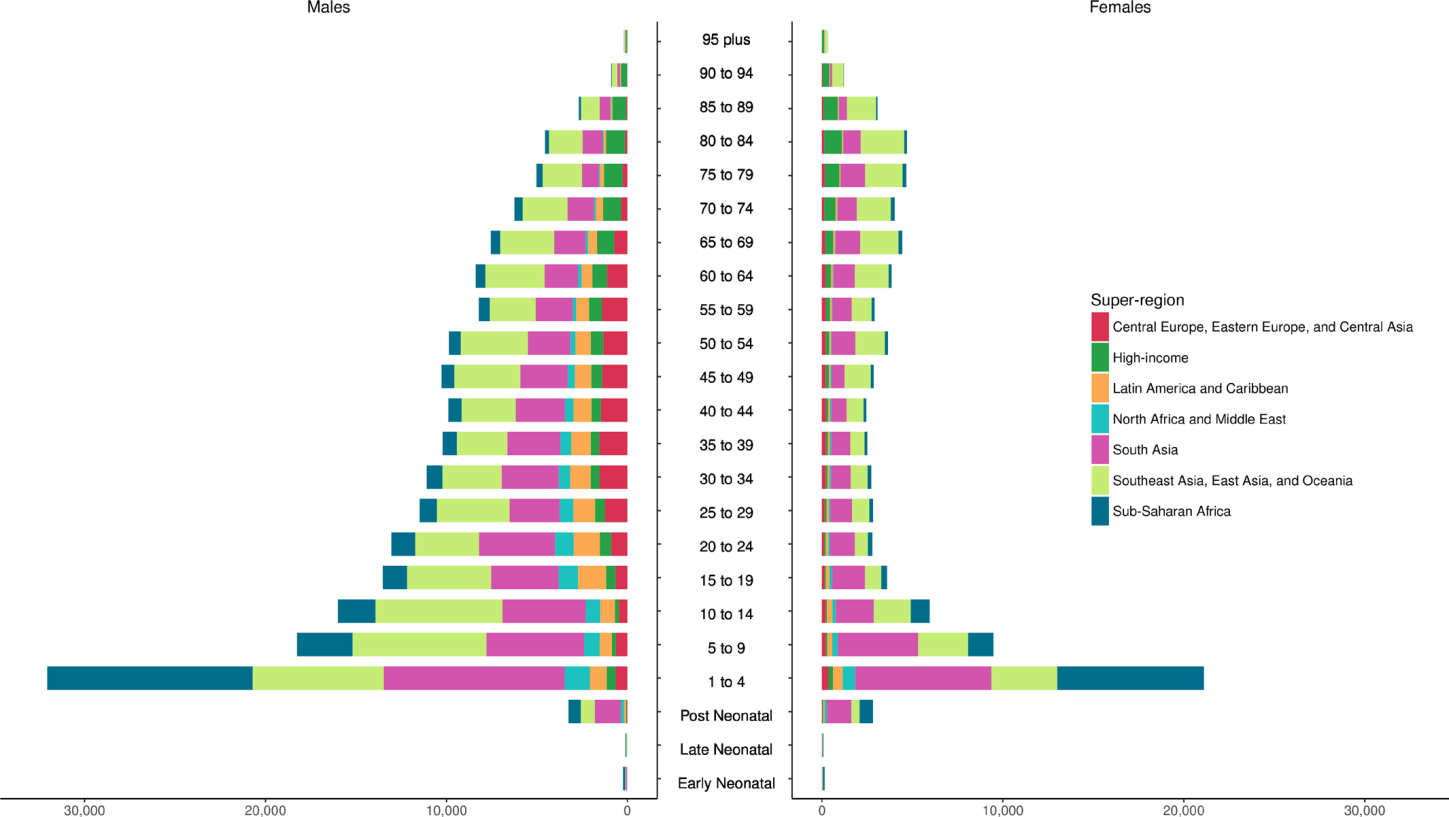
Incidence of unintentional drowning mortality by age group, sex and super-region in 2017.

The age-standardised cause-specific mortality rates for unintentional drowning per 100 000 for each country in 2017 by sex are shown in figure 1, with male rates generally higher than female rates. Figure 2 shows all-age death counts for unintentional drowning for each country in 2017 by sex, with China, India, Pakistan and Bangladesh all having over 10 000 deaths in 2017. Figure 3 shows the number of deaths by age, sex and super-region, with children 1–4 years having the highest number of deaths. For females, there is an increase in the number of deaths in the 60–84 years age groups.

**Figure 4:**
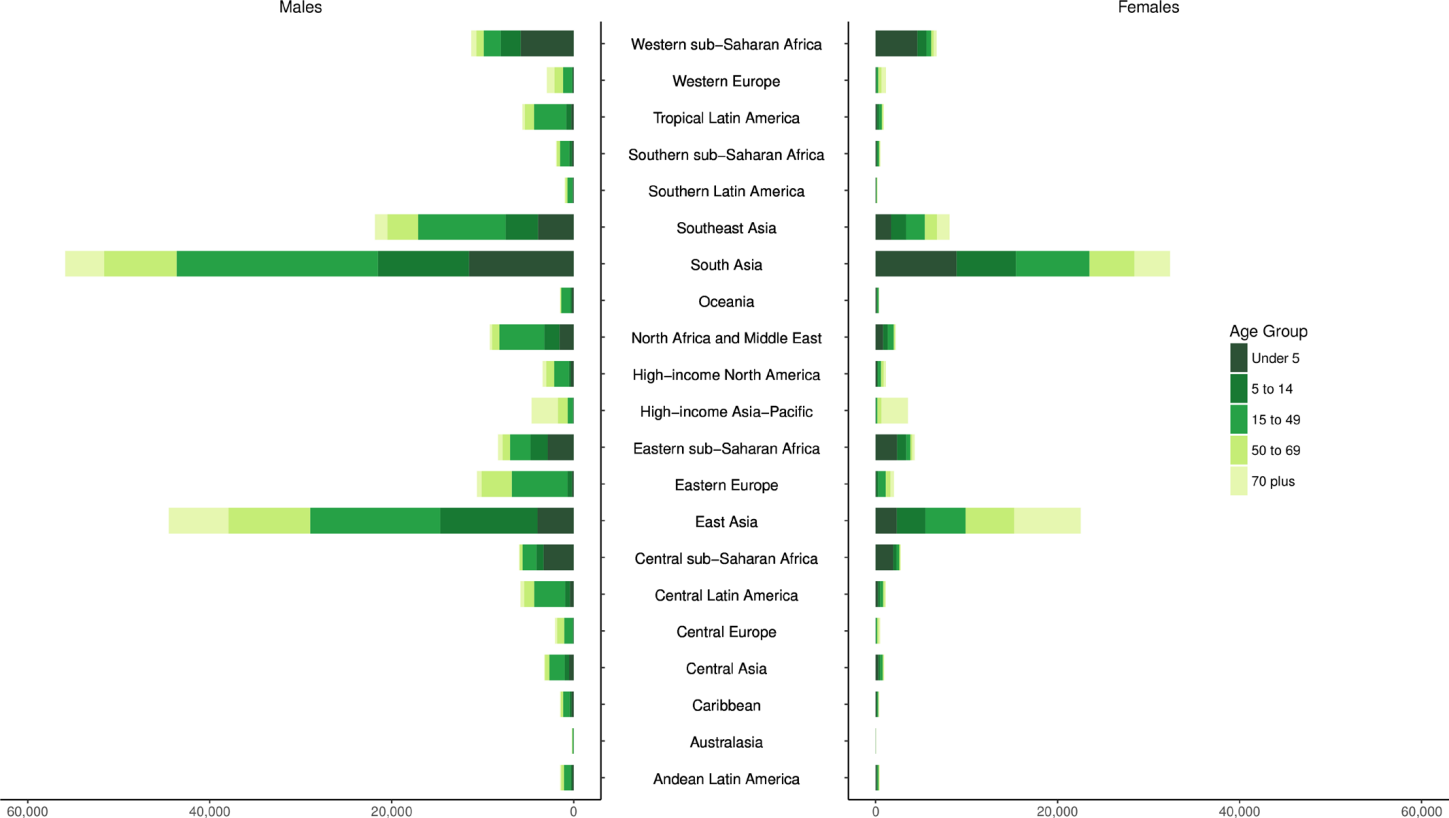
Incidence of unintentional drowning mortality by region, sex and five age groups in 2017.

The distributions of deaths by region for five age groups (under 5, 5–14, 15–49, 50–69 and 70 plus) are shown in figure 4. These figures emphasise how there is greater unintentional drowning mortality in males than females in most age groups, with sex differences less pronounced in certain regions such as South Asia (online supplementary table S1). Distributions of drowning deaths by age vary depending on region. For example, there are relatively more deaths from unintentional drowning in younger age groups in lower-income regions, compared with other age groups in those regions, such as the sub-Saharan Africa, South Asia, North Africa, Middle East and Central Asia regions.

### Years of life lost

**Figure 5:**
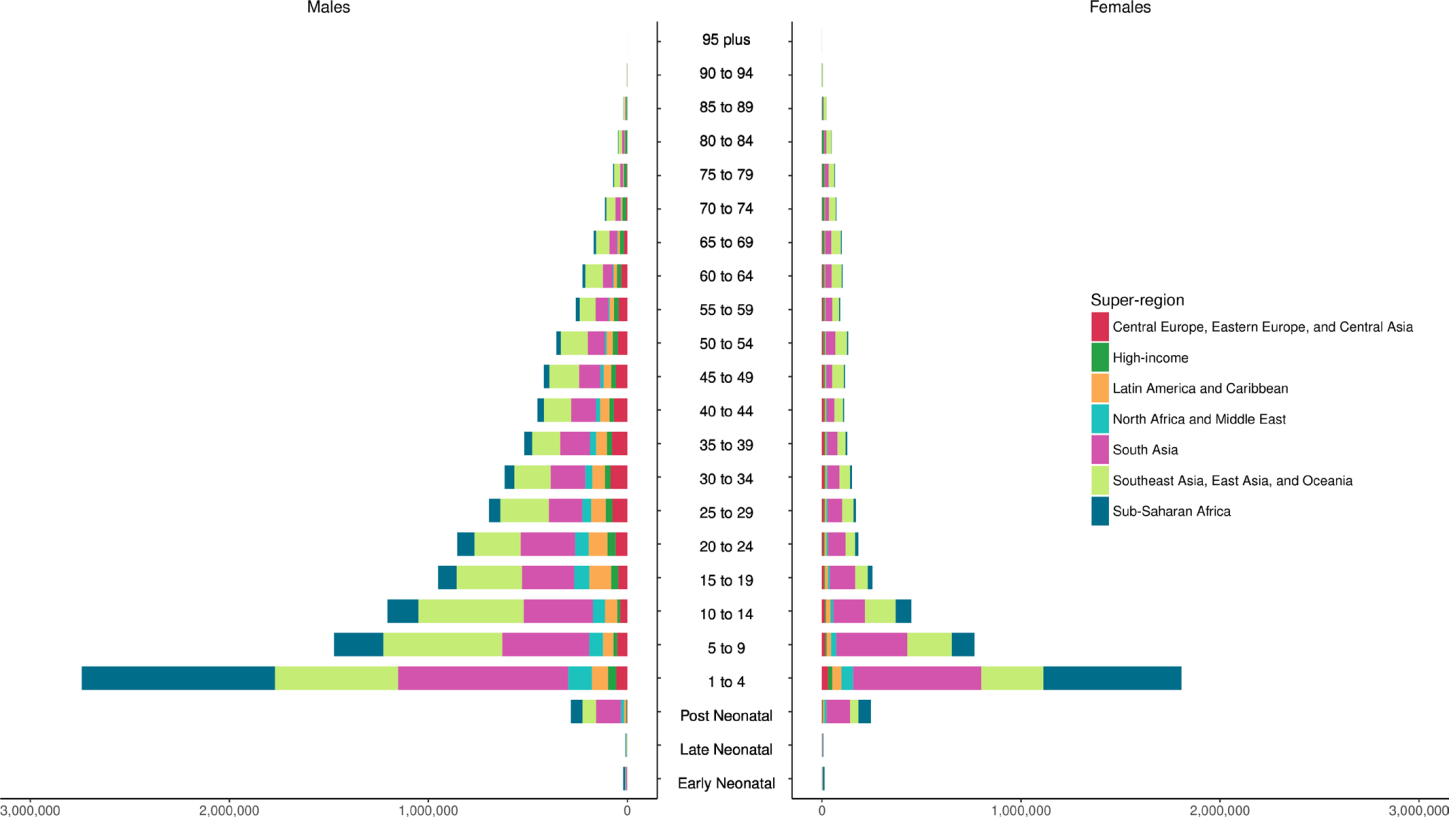
Years of life lost to unintentional drowning by age group, sex and super-region in 2017.

**Figure 6:**
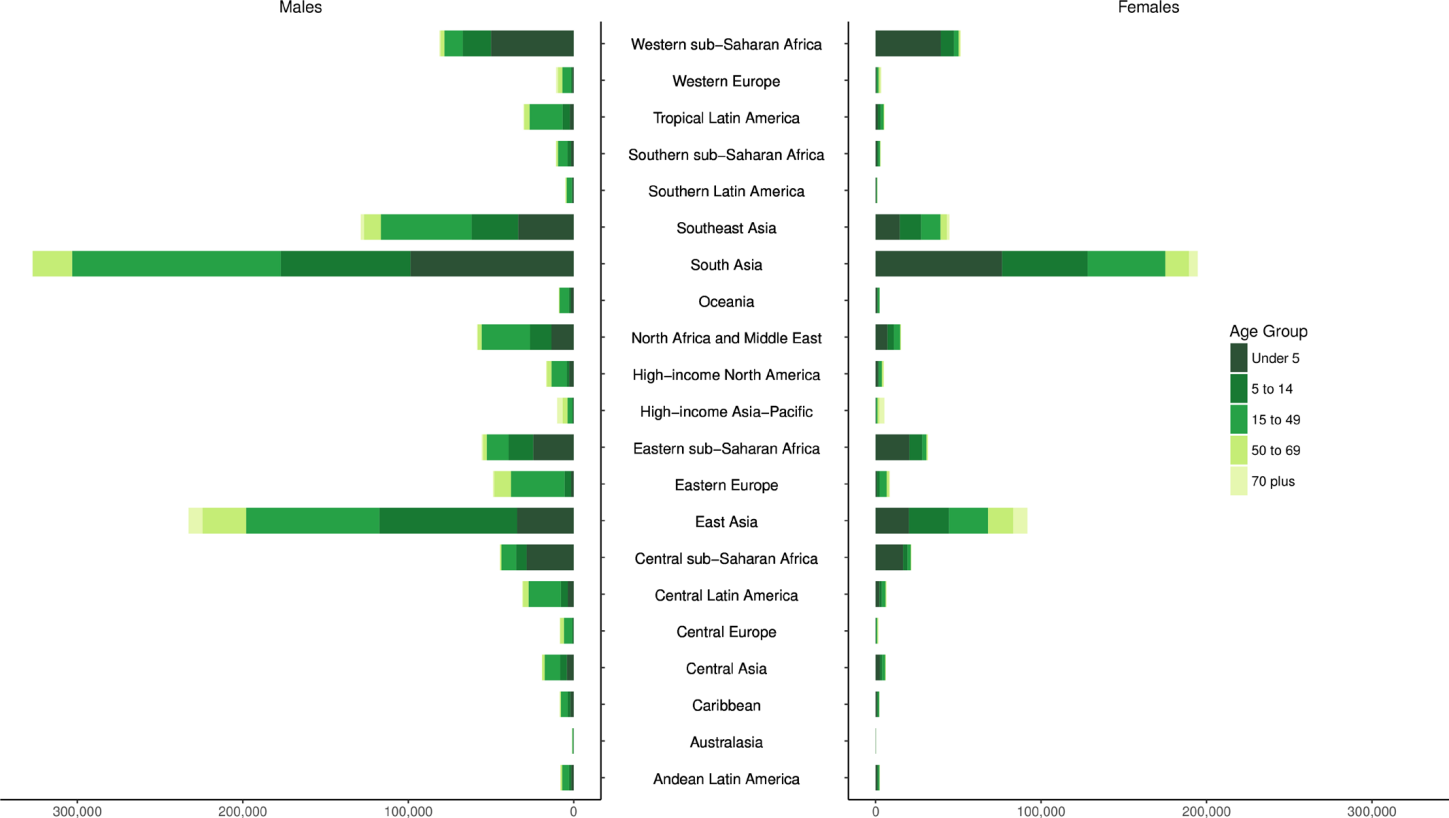
Years of life lost to unintentional drowning by region, sex and five age groups, 2017.

YLLs by super-region, sex and age group are shown in figure 5 and YLLs by sex, region and five age groups (under 5, 5–14, 15–49, 50–69 and 70 plus) are shown in figure 6, with Asia (South, East and Southeast) having the highest YLLs. In 1990, drowning caused 37 925 314 (UI: 33 968 621 to 41 237 399) total YLLs, a number which decreased by 56.3% (UI: 51.2% to 59.7%) to a total of 16 563 278 (UI: 15 784 185 to 17 349 952) YLLs in 2017. The age-standardised YLL rate in 1990 was 632.3 (UI: 568.8 to 685.6) per 100 000, which decreased by 63.9% (UI: 59.8% to 66.6%) to 228.3 (UI: 217.2 to 239.7) YLLs per 100 000 in 2017. South Asia was the region with the greatest number of YLLs in 2017, with 5 273 794 (UI: 4 819 285 to 5 744 698) YLLs across both sexes and all ages. Oceania, however, was the region with the highest rate of age-standardised YLLs in 2017, with 828.2 (UI: 672.7 to 997.9) YLLs per 100 000 across both sexes (online supplementary table S2).

### Socio-demographic Index

**Figure 7:**
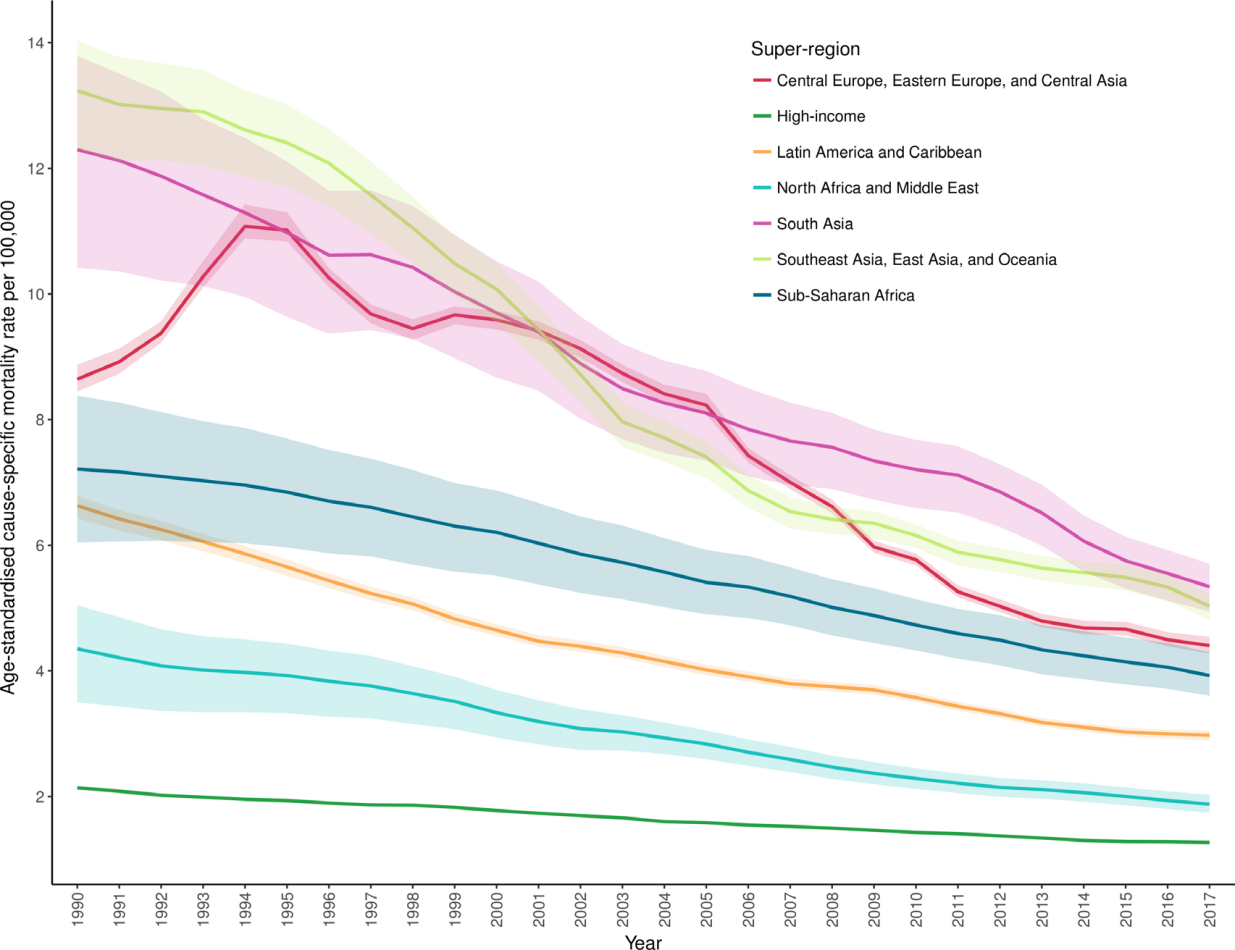
Age-standardised cause-specific mortality rate per 100 000 for unintentional drowning by year and super-region (1990–2017).

The trends over time in terms of age-standardised cause-specific mortality rates from drowning for each super-region in GBD between 1990 and 2017 are shown in figure 7, with decreases seen across all super-regions. The numbers of deaths and age-standardised cause-specific mortality rates were noted to decrease in every SDI quintile and for most countries. In 27 countries, mainly in the Oceania region, there was no significant percentage change between 1990 and 2017 for age-standardised cause-specific mortality rates. Countries outside of the Oceania region that did not experience decreases in age-standardised cause-specific mortality rates were Lesotho, Zimbabwe and Cape Verde. Selected countries with large populations were noted to still have large numbers of deaths from unintentional drowning in 2017, specifically China, India, Bangladesh and Pakistan, accounting for just over half of all drowning deaths (51.2%) (online supplementary table S1).

## Discussion

There were approximately 295 210 (UI: 284 493 to 3 06 187) unintentional drowning deaths in 2017, an almost 50% reduction (44.5%) in unintentional drowning deaths over the last 28 years. This study explored changes between 1990 and 2017 in unintentional drowning mortality across the globe, and while there has been an expected reduction in unintentional drowning mortality, as has been seen in other studies,^7 24^ the reduction was not uniform. Countries from middle SDI groupings had the greatest reduction (54.0%), again indicating that urbanisation and development are possible drivers of the decrease in drowning deaths; however, other drivers, such as greater investment in water safety, government recognition of the issue, changing social norms or coding frameworks, could be contributing.^1 2 14^

Four countries in 2017 (China, India, Pakistan and Bangladesh) accounted for half of all drowning deaths, with most countries experiencing a decrease in drowning rates and numbers between 1990 and 2017. All four countries in 2017 had rates higher than the global average of 4.0 (3.6–4.1) and while Bangladesh had experienced the largest reduction in the drowning death rate (a reduction of 70.4%) it had a rate of 9.5 (7.6–12.1). The exception was Oceania, which saw a near doubling (80.1% increase) of drowning fatalities; however, it is unclear what is the cause of this increase. Such trends represent an ongoing challenge for those working in drowning prevention in the Oceania region.

Children, residents of countries in Asia and Africa, males, and low-SDI and middle-SDI countries account for the majority of drowning deaths. Unintentional drowning is preventable and is linked to exposure.^6 25–27^ Increasing economic prosperity and urbanisation has brought about safer domestic and work-related conditions, with increasing exposure around recreational use of water.^7 16 28–30^ Children, with their natural curiosity, evolving cognition and lack of swimming skills, remain over-represented in unintentional drowning statistics. This is highlighted by the high number of YLLs from drowning.

Child drowning prevention is a well-established area of research,^18 31^ with multiple studies identifying strategies which can effectively prevent unintentional drowning in high-income countries and LMICs, including restricting access to water,^32^ supervision,^33^ learning to swim,^34^ crèches^35^ and the use of life jackets.^36^ However, it is noted that there is a need to take interventions to scale^18^ and that strategies will need to take into account factors such as age, gender and access to safe aquatic locations.^37^ Cardiopulmonary resuscitation (CPR) is also a potentially lifesaving procedure capable of preventing fatal drowning, rather than preventing the initial drowning incident, and needs to be taught to supervisors of people in and around water, including parents.^38^ It also appears that while each strategy (ie, restricting access to water, supervision, learning to swim, use of lifejackets and CPR) is helpful, the best results are seen when all measures, combined, are enacted.^33^ Although a range of other strategies have been proposed (such as alarms, cameras and child behaviour modification), there is not as strong an evidence base for these interventions to date.

WHO, in recognising unintentional drowning as a public health threat, has recently developed an implementation guide to help countries address the challenge of unintentional drowning.^3^ This guide is designed to ensure that each country understands the factors contributing to unintentional drowning and has a set of strategies that can support unintentional drowning prevention. It is vital that all countries have quality data (comprehensive, up-to-date, accurate and useable for planning) at national and subnational levels to inform a context-specific and evidence-based understanding of the burden of unintentional drowning. Presenting the mortality rate of unintentional drowning across the globe can help countries benchmark their unintentional drowning rate against that of similar countries (by size, location, SDI, etc) as well as provide an opportunity to discuss what might be reasonable unintentional drowning reduction targets to aim for in the development of a national water safety plan, as recommended by WHO.^3^ This information can be used to help evaluate the success at a country level of a comprehensive unintentional drowning prevention strategy.

While reducing drowning in low-SDI and middle-SDI countries is a challenge, recent work in Bangladesh^34^ and Thailand^29 39^ has demonstrated that country-level commitment and appropriate resourcing to unintentional drowning reduction can have a marked impact on mortality and morbidity rates. A recent review^5^ of drowning in LMICs found only a small number of studies from LMICs (n=62 studies) which met their inclusion criteria, noting that data used in a third of the included studies were from autopsy and medico-legal records, with the rest from surveys and interviews, hospital records, media and ambulance records. This highlights the paucity of published data on drowning from LMICs, compounded by a lack of medico-legal, uniformly collected, comprehensive data. Strengthening drowning data (monitoring) in LMICs should be prioritised, leading to an increase in published studies, thereby enhancing the evidence base of drowning epidemiology and risk factors to better inform prevention efforts. The limited peer-reviewed studies from Africa also highlight the need for a greater focus on drowning in Africa, especially considering that 11 of the 16 articles were from South Africa.^5^ Another review of drowning in South Africa showed large heterogeneity in drowning across the country, a potential challenge for estimating the number of drowning deaths in Africa.^13^

Future studies should focus analysis in regions and countries with high rates of drowning mortality, particularly LMICs, as well as explore seasonal variations in drowning, the impact of alcohol, and cultural reasons for differing risks of drowning. The impact of a country’s development index on drowning mortality rates, in addition to SDI, could be examined in future studies.

### Limitations

This study used data from the GBD project, combining a range of data sources to provide the most comprehensive picture of unintentional drowning mortality across the globe. However, it is not without its limitations. The use of mutually exclusive ICD codes means that not all drowning deaths are captured in the original data and recent work has shown that drowning may not be the primarily coded cause of death, and while GBD redistribution methods should account for miscoding, more research and validation is required to continue improving data quality.^14 15^ This study included unintentional drowning deaths only, and as such does not include intentional drowning, drowning following aquatic transport or other transportation incidents, or drowning due to natural disasters. Additionally, in countries, especially LMICs, where there is not a regular collection of death data or which lack hospital data, there is potential for misestimation of drowning deaths, which is an ongoing area of GBD research.^40^

This study captures greater uncertainty in data-sparse areas, and this should be viewed alongside the point in time estimates. As drowning is not evenly distributed across countries, this study used models associated with drowning, such as countries with greater populations residing within 10 km of the coast, with high rainfall, and those close to sea level (table 1). These assumptions and covariates will benefit from ongoing review and refinement. For example, some landlocked countries may have more exposure to drowning in inland waterways such as rivers and lakes.^41^ This study did not explore the circumstances leading to drowning, aquatic location where the incident occurred, or the impact of alcohol, which is a significant contributor to drowning.^42–44^ There is also further work required to quantify the burden of drowning among migrants. Future drowning studies should also encompass all drowning deaths (ie, transport related, disaster related, unintentional, intentional and undetermined intent).

## Conclusion

Unintentional drowning deaths across the globe continue to decrease, with the 2017 GBD (data from all national states) estimates showing 295 210 drowning deaths. The reduction in unintentional drowning has not been consistent, with some countries achieving greater reductions than others. Future GBD studies should evaluate actions at a country level to inform future prevention efforts. Children, especially those from LMICs had higher fatal unintentional drowning rates, and achieving reductions in drowning in China, India, Bangladesh and Pakistan should be prioritised due to their population size. There is a need for investment in drowning prevention strategies, policy and research, including the evaluation of preventive strategies to continue the downward trend in drowning rates seen in this study.

**What is already known on the subject**

- Mortality codes for unintentional drowning only capture 40%–50% of drowning deaths in high-income countries.
- Limited information exists on drowning in low-income and middle-income countries, especially in Latin America and Africa.
- Drowning is a public health challenge requiring further research and sustained investment in evidence-based interventions.

**What this study adds**

- Almost 300 000 people died from unintentional drowning in 2017, exclusive of drowning in natural disasters and due to transport incidents.
- The global age-standardised mortality rate from unintentional drowning declined by 57% from 1990 to 2017.
- Trends over time by geographical regions and Socio-demographic Index, from 1990 to 2017. Half (51.2%) of unintentional drowning-related deaths in 2017 occurred in China, India, Pakistan and Bangladesh.
