# Correction: *The burden of unintentional drowning: Global, regional and national estimates of mortality from the Global Burden of Disease 2017 Study*

**DOI:** 10.1136/injuryprev-2019-043484corr1

**Published:** 2020-09-28

Franklin RC, Peden AE, Hamilton EB, *et al*. The burden of unintentional drowning: global, regional and national estimates of mortality from the Global Burden of Disease 2017 Study. *Inj Prev* 2020;1–13. doi: 10.1136/injuryprev-2019-043484.

This article was previously published with errors in authorship and affiliations. Please note the below updates:

The updated affiliations for Rakhi Dandona^4,5,39^ are

4 Institute for Health Metrics and Evaluation, University of Washington, Seattle, Washington, USA.

5 Department of Health Metrics Sciences, School of Medicine, University of Washington, Seattle, Washington, USA.

39 Public Health Foundation of India, Gurugram, India.

Author Hai Quang Pham has been added prior to Suzanne Polinder. The affiliation for Hai Quang Pham^107^ is

107 Institute for Global Health Innovations, Duy Tan University, Hanoi, Vietnam

Author Vafa Rahimi-Movaghar has been added prior to Saleem Muhammad Rana. The affiliation for Vafa Rahimi-Movaghar^123^ is

123 Sina Trauma and Surgery Research Centre, Tehran University of Medical Sciences, Tehran, Iran

Author Bach Xuan Tran has been added prior to Pascual R Valdez. The affiliation for Bach Xuan Tran^151^ is

151 Department of Health Economics, Hanoi Medical University, Hanoi, Vietnam.

This affiliation has been added in the affiliation list.

