# Supplementary material for: The burden of unintentional drowning: global, regional and national estimates of mortality from the Global Burden of Disease 2017 Study
Source: Inj Prev. 2020 Feb 20;26(Suppl 1):i83–95. doi: 10.1136/injuryprev-2019-043484 (PMC7571364; doi:10.1136/injuryprev-2019-043484)
Supplement: Supplementary data [file injuryprev-2019-043484supp003.pdf]

Table 1: Cause-specific mortality in and percentage change between 1990 and 2017 for drowning

Location	All-age mortality counts (95% UI)			Age-standardised cause-specific mortality rates per 100,000 (95% UI)		
	1990	2017	Percentage change between 1990 and 2017	1990	2017	Percentage change between 1990 and 2017
**Global**	**531 956 (484 107 to 572 854)**	**295 210 (284 493 to 306 187)**	**-44.5 (-48.3 to -38.9)**	**9.3 (8.5 to 10.0)**	**4.0 (3.8 to 4.1)**	**-57.4 (-60.1 to -53.3)**
**Low SDI**	**117 837 (92 982 to 137 713)**	**67 961 (62 168 to 74 278)**	**-42.3 (-51.0 to -26.1)**	**13.2 (10.8 to 15.1)**	**5.4 (4.9 to 5.8)**	**-59.3 (-64.7 to -49.6)**
**Low-middle SDI**	**109 554 (93 210 to 122 877)**	**74 477 (69 932 to 79 856)**	**-32.0 (-39.6 to -20.8)**	**9.3 (8.1 to 10.3)**	**4.6 (4.3 to 4.9)**	**-51.0 (-55.9 to -44.3)**
**Middle SDI**	**189 203 (175 772 to 200 765)**	**87 094 (83 787 to 90 377)**	**-54.0 (-56.7 to -50.8)**	**11.5 (10.7 to 12.2)**	**4.4 (4.2 to 4.5)**	**-62.0 (-64.0 to -59.7)**
**High-middle SDI**	**88 348 (84 729 to 92 590)**	**44 286 (42 893 to 45 645)**	**-49.9 (-52.2 to -47.3)**	**8.0 (7.7 to 8.4)**	**3.2 (3.1 to 3.3)**	**-60.2 (-62.0 to -58.2)**
**High SDI**	**23 773 (23 497 to 24 094)**	**20 081 (19 699 to 20 515)**	**-15.5 (-17.3 to -13.7)**	**2.5 (2.4 to 2.5)**	**1.3 (1.3 to 1.3)**	**-46.9 (-48.1 to -45.7)**
**Central Europe, Eastern Europe, and Central Asia**	**36 096 (35 245 to 37 051)**	**19 360 (18 857 to 19 935)**	**-46.4 (-47.7 to -45.0)**	**8.6 (8.4 to 8.9)**	**4.4 (4.3 to 4.5)**	**-49.1 (-50.5 to -47.7)**
**Central Asia**	**7 686 (7 398 to 8 038)**	**4 177 (3 944 to 4 423)**	**-45.7 (-49.4 to -41.9)**	**10.3 (10.0 to 10.7)**	**4.6 (4.3 to 4.8)**	**-55.5 (-58.4 to -52.7)**
Armenia	257 (241 to 275)	48 (45 to 51)	-81.4 (-83.2 to -79.2)	7.4 (7.0 to 7.9)	1.6 (1.5 to 1.7)	-78.7 (-80.9 to -76.2)
Azerbaijan	573 (524 to 637)	308 (265 to 354)	-46.4 (-54.4 to -37.8)	7.4 (6.8 to 8.2)	3.1 (2.7 to 3.6)	-58.3 (-64.4 to -51.6)
Georgia	435 (406 to 463)	136 (126 to 147)	-68.8 (-71.8 to -65.5)	7.9 (7.3 to 8.4)	3.4 (3.2 to 3.7)	-56.3 (-60.6 to -51.3)
Kazakhstan	2 072 (1 935 to 2 197)	923 (836 to 1 023)	-55.4 (-60.0 to -50.3)	12.0 (11.2 to 12.6)	5.1 (4.6 to 5.6)	-57.7 (-62.0 to -53.2)
Kyrgyzstan	646 (596 to 705)	285 (259 to 314)	-55.9 (-61.3 to -49.7)	13.9 (12.9 to 15.0)	4.5 (4.1 to 4.9)	-67.5 (-70.9 to -63.6)
Mongolia	150 (123 to 182)	143 (122 to 165)	-4.6 (-21.9 to 17.1)	6.2 (5.3 to 7.3)	4.3 (3.7 to 4.9)	-31.0 (-42.3 to -17.1)
Tajikistan	713 (643 to 805)	711 (627 to 814)	-0.3 (-15.7 to 18.6)	11.4 (10.5 to 12.6)	7.3 (6.5 to 8.2)	-36.3 (-44.9 to -25.9)
Turkmenistan	511 (442 to 588)	153 (134 to 180)	-70.0 (-75.7 to -62.7)	11.2 (10.0 to 12.6)	3.0 (2.6 to 3.5)	-73.2 (-77.7 to -67.4)
Uzbekistan	2 330 (2 102 to 2 589)	1 471 (1 306 to 1 668)	-36.9 (-46.7 to -25.8)	9.5 (8.7 to 10.3)	4.6 (4.1 to 5.2)	-51.8 (-58.6 to -44.1)
**Central Europe**	**5 442 (5 322 to 5 561)**	**2 532 (2 430 to 2 645)**	**-53.5 (-55.4 to -51.6)**	**4.4 (4.3 to 4.5)**	**1.9 (1.8 to 2.0)**	**-57.6 (-59.4 to -55.7)**
Albania	145 (128 to 160)	38 (31 to 45)	-74.0 (-79.3 to -67.4)	4.0 (3.6 to 4.4)	1.4 (1.2 to 1.7)	-64.6 (-71.7 to -56.0)
Bosnia and Herzegovina	50 (41 to 58)	35 (31 to 39)	-28.7 (-45.6 to -12.1)	1.1 (0.9 to 1.3)	0.9 (0.8 to 1.0)	-18.6 (-35.0 to 0.3)
Bulgaria	333 (318 to 347)	145 (133 to 157)	-56.5 (-60.3 to -52.7)	3.8 (3.6 to 4.0)	1.8 (1.7 to 2.0)	-52.5 (-57.1 to -47.7)
Croatia	149 (142 to 156)	83 (77 to 90)	-44.4 (-49.1 to -38.9)	2.8 (2.7 to 3.0)	1.5 (1.3 to 1.6)	-48.8 (-53.3 to -43.6)
Czech Republic	327 (313 to 341)	193 (179 to 209)	-40.8 (-45.7 to -35.8)	3.1 (3.0 to 3.3)	1.4 (1.3 to 1.5)	-55.0 (-59.0 to -50.8)
Hungary	342 (328 to 359)	130 (121 to 140)	-62.1 (-65.2 to -58.7)	3.2 (3.1 to 3.4)	1.1 (1.1 to 1.2)	-64.2 (-67.4 to -60.9)
Macedonia	30 (27 to 33)	26 (24 to 29)	-11.9 (-23.1 to 1.0)	1.5 (1.4 to 1.7)	1.1 (1.0 to 1.2)	-26.3 (-35.8 to -14.9)
Montenegro	9 (8 to 10)	6 (5 to 7)	-35.3 (-45.3 to -24.1)	1.5 (1.3 to 1.7)	0.8 (0.7 to 0.9)	-43.0 (-51.9 to -33.0)
Poland	1 819 (1 752 to 1 891)	904 (835 to 980)	-50.3 (-54.4 to -45.6)	4.7 (4.5 to 4.9)	2.0 (1.8 to 2.1)	-58.1 (-61.7 to -54.0)
Romania	1 721 (1 664 to 1 781)	714 (665 to 773)	-58.5 (-61.6 to -55.1)	7.4 (7.1 to 7.7)	3.2 (2.9 to 3.4)	-57.1 (-60.4 to -53.4)
Serbia	264 (239 to 293)	114 (104 to 124)	-57.0 (-62.2 to -51.1)	2.9 (2.6 to 3.2)	1.1 (1.0 to 1.2)	-63.3 (-68.1 to -57.9)
Slovakia	210 (193 to 226)	122 (111 to 136)	-41.8 (-48.6 to -33.6)	3.9 (3.6 to 4.2)	1.9 (1.7 to 2.1)	-51.3 (-57.1 to -44.0)
Slovenia	44 (42 to 47)	22 (20 to 24)	-50.9 (-55.5 to -46.2)	2.1 (2.0 to 2.2)	0.8 (0.7 to 0.8)	-62.8 (-66.6 to -59.0)
**Eastern Europe**	**22 968 (22 181 to 23 692)**	**12 651 (12 304 to 13 036)**	**-44.9 (-46.6 to -43.3)**	**10.1 (9.7 to 10.4)**	**5.4 (5.3 to 5.6)**	**-46.2 (-47.9 to -44.5)**
Belarus	1 278 (1 223 to 1 340)	630 (572 to 689)	-50.7 (-55.4 to -45.4)	12.3 (11.7 to 13.0)	5.8 (5.2 to 6.3)	-53.2 (-58.0 to -48.2)
Estonia	215 (205 to 224)	48 (42 to 55)	-77.7 (-80.7 to -74.4)	13.6 (13.0 to 14.3)	3.1 (2.7 to 3.5)	-77.5 (-80.5 to -74.1)
Latvia	440 (422 to 458)	142 (124 to 162)	-67.8 (-72.0 to -63.0)	16.1 (15.4 to 16.8)	6.2 (5.4 to 7.0)	-61.6 (-66.6 to -55.9)
Lithuania	550 (528 to 573)	214 (197 to 234)	-61.1 (-64.4 to -57.5)	14.4 (13.8 to 15.0)	6.1 (5.6 to 6.6)	-57.8 (-61.4 to -53.8)
Moldova	530 (502 to 558)	208 (195 to 222)	-60.7 (-63.8 to -57.4)	11.9 (11.3 to 12.6)	5.1 (4.7 to 5.5)	-57.4 (-60.9 to -53.7)
Russia	13 638 (12 962 to 14 141)	8 259 (8 051 to 8 495)	-39.4 (-41.2 to -37.0)	8.9 (8.5 to 9.2)	5.1 (4.9 to 5.2)	-43.0 (-44.7 to -41.1)
Ukraine	6 316 (6 061 to 6 604)	3 150 (2 938 to 3 377)	-50.1 (-54.1 to -45.8)	12.1 (11.6 to 12.6)	6.5 (6.1 to 7.0)	-46.0 (-50.4 to -41.2)
**High-income**	**19 448 (19 196 to 19 724)**	**18 253 (17 880 to 18 645)**	**-6.1 (-8.2 to -3.7)**	**2.1 (2.1 to 2.2)**	**1.3 (1.2 to 1.3)**	**-40.8 (-42.2 to -39.1)**
**Australasia**	**372 (357 to 391)**	**269 (244 to 296)**	**-27.7 (-35.0 to -19.5)**	**1.9 (1.8 to 2.0)**	**0.9 (0.8 to 1.0)**	**-51.8 (-57.0 to -45.9)**
Australia	300 (284 to 316)	215 (192 to 242)	-28.1 (-36.8 to -18.5)	1.9 (1.8 to 2.0)	0.9 (0.8 to 1.0)	-53.2 (-59.2 to -46.4)
New Zealand	73 (69 to 77)	54 (50 to 57)	-26.3 (-32.6 to -19.9)	2.2 (2.1 to 2.3)	1.2 (1.1 to 1.3)	-44.2 (-49.2 to -39.0)
**High-income Asia-Pacific**	**6 257 (6 099 to 6 414)**	**8 160 (7 890 to 8 460)**	**30.4 (25.2 to 36.5)**	**3.7 (3.6 to 3.8)**	**2.0 (2.0 to 2.1)**	**-45.1 (-47.5 to -42.3)**
Brunei	20 (18 to 23)	19 (17 to 21)	-8.1 (-22.4 to 8.9)	8.8 (7.9 to 9.7)	4.5 (4.0 to 5.1)	-48.3 (-55.9 to -39.3)
Japan	3 577 (3 516 to 3 635)	7 371 (7 132 to 7 639)	106.1 (99.3 to 114.6)	2.6 (2.6 to 2.7)	2.3 (2.2 to 2.3)	-14.0 (-17.0 to -10.8)
South Korea	2 595 (2 454 to 2 739)	743 (674 to 821)	-71.4 (-74.3 to -68.0)	6.0 (5.7 to 6.3)	1.1 (1.0 to 1.2)	-81.1 (-83.0 to -78.9)
Singapore	65 (62 to 69)	26 (24 to 28)	-59.7 (-63.2 to -55.9)	2.7 (2.5 to 2.8)	0.4 (0.4 to 0.5)	-83.4 (-84.8 to -81.8)
**High-income North America**	**5 002 (4 919 to 5 104)**	**4 547 (4 396 to 4 717)**	**-9.1 (-13.1 to -5.1)**	**1.8 (1.8 to 1.9)**	**1.2 (1.2 to 1.3)**	**-32.9 (-35.9 to -29.9)**
Canada	421 (403 to 440)	325 (301 to 350)	-22.8 (-29.2 to -15.5)	1.6 (1.5 to 1.7)	0.9 (0.8 to 0.9)	-45.4 (-50.6 to -39.7)
Greenland	8 (7 to 10)	4 (3 to 4)	-56.3 (-64.5 to -48.6)	14.8 (13.2 to 17.3)	6.0 (5.4 to 6.7)	-59.3 (-66.9 to -52.1)
USA	4 573 (4 489 to 4 669)	4 218 (4 069 to 4 385)	-7.7 (-11.9 to -3.6)	1.9 (1.8 to 1.9)	1.3 (1.2 to 1.3)	-31.6 (-34.9 to -28.3)
**Southern Latin America**	**1 875 (1 806 to 1 940)**	**1 204 (1 108 to 1 320)**	**-35.8 (-41.2 to -29.0)**	**3.8 (3.6 to 3.9)**	**1.8 (1.7 to 2.0)**	**-52.3 (-56.3 to -47.4)**
Argentina	1 202 (1 142 to 1 259)	710 (630 to 802)	-40.9 (-47.9 to -32.3)	3.6 (3.4 to 3.8)	1.6 (1.4 to 1.8)	-55.6 (-60.7 to -49.3)
Chile	505 (478 to 532)	402 (351 to 459)	-20.3 (-31.5 to -7.6)	3.9 (3.7 to 4.1)	2.1 (1.8 to 2.4)	-47.0 (-54.1 to -38.8)
Uruguay	169 (161 to 177)	92 (81 to 104)	-45.4 (-52.5 to -37.8)	5.3 (5.1 to 5.6)	2.6 (2.3 to 2.9)	-51.7 (-57.8 to -44.7)
**Western Europe**	**5 941 (5 840 to 6 039)**	**4 073 (3 934 to 4 205)**	**-31.4 (-34.2 to -28.8)**	**1.5 (1.4 to 1.5)**	**0.7 (0.7 to 0.7)**	**-51.4 (-53.6 to -49.5)**
Andorra	1 (0 to 1)	0 (0 to 1)	-15.2 (-33.9 to 9.1)	1.0 (0.8 to 1.3)	0.5 (0.4 to 0.6)	-52.6 (-64.0 to -37.6)
Austria	120 (114 to 127)	66 (61 to 72)	-44.9 (-50.0 to -39.3)	1.5 (1.4 to 1.6)	0.6 (0.5 to 0.6)	-61.6 (-65.8 to -57.1)
Belgium	139 (132 to 146)	93 (86 to 100)	-33.0 (-38.6 to -26.6)	1.3 (1.2 to 1.4)	0.6 (0.6 to 0.7)	-51.3 (-56.0 to -46.0)
Cyprus	27 (24 to 30)	25 (22 to 28)	-7.1 (-20.0 to 8.9)	3.3 (2.9 to 3.6)	1.5 (1.4 to 1.7)	-53.1 (-59.4 to -45.1)

Location	All-age mortality counts (95% UI)			Age-standardised cause-specific mortality rates per 100,000 (95% UI)		
	1990	2017	Percentage change between 1990 and 2017	1990	2017	Percentage change between 1990 and 2017
Denmark	47 (44 to 50)	34 (32 to 37)	-26.8 (-33.6 to -19.4)	0.9 (0.8 to 1.0)	0.5 (0.4 to 0.5)	-46.4 (-52.4 to -39.9)
Finland	173 (165 to 181)	116 (107 to 127)	-32.6 (-38.9 to -25.5)	3.1 (2.9 to 3.2)	1.5 (1.4 to 1.6)	-50.8 (-55.4 to -45.6)
France	1 135 (1 092 to 1 180)	1 042 (972 to 1 112)	-8.2 (-15.0 to -0.9)	1.8 (1.8 to 1.9)	1.2 (1.1 to 1.3)	-34.7 (-40.1 to -29.0)
Germany	1 041 (998 to 1 089)	553 (498 to 617)	-46.9 (-52.6 to -40.3)	1.4 (1.3 to 1.5)	0.5 (0.5 to 0.6)	-63.2 (-67.7 to -58.3)
Greece	283 (272 to 294)	359 (333 to 385)	27.0 (17.1 to 37.7)	2.3 (2.2 to 2.4)	2.1 (2.0 to 2.3)	-9.1 (-16.6 to -1.1)
Iceland	4 (4 to 4)	4 (3 to 4)	-12.7 (-21.4 to -3.5)	1.6 (1.5 to 1.7)	0.9 (0.9 to 1.0)	-41.0 (-47.4 to -34.2)
Ireland	75 (71 to 79)	45 (40 to 49)	-40.0 (-46.8 to -33.4)	2.0 (1.9 to 2.2)	0.8 (0.7 to 0.9)	-59.8 (-64.5 to -55.1)
Israel	72 (68 to 77)	53 (49 to 58)	-26.5 (-34.0 to -18.0)	1.4 (1.4 to 1.5)	0.6 (0.5 to 0.6)	-60.2 (-64.2 to -55.5)
Italy	683 (657 to 710)	437 (406 to 468)	-36.1 (-41.5 to -30.6)	1.1 (1.1 to 1.2)	0.6 (0.5 to 0.6)	-48.8 (-53.6 to -43.7)
Luxembourg	4 (4 to 5)	3 (3 to 4)	-23.8 (-33.3 to -12.9)	1.1 (1.0 to 1.1)	0.5 (0.4 to 0.5)	-56.4 (-62.2 to -50.0)
Malta	5 (4 to 5)	5 (4 to 5)	1.5 (-8.0 to 12.0)	1.2 (1.1 to 1.3)	0.9 (0.8 to 1.0)	-29.3 (-36.4 to -20.8)
Netherlands	134 (125 to 145)	108 (100 to 116)	-19.6 (-27.5 to -11.2)	1.0 (0.9 to 1.1)	0.5 (0.5 to 0.5)	-50.5 (-56.9 to -44.0)
Norway	94 (91 to 96)	56 (54 to 59)	-39.6 (-42.7 to -36.5)	2.1 (2.0 to 2.1)	0.9 (0.8 to 0.9)	-58.5 (-61.0 to -56.3)
Portugal	257 (243 to 270)	121 (112 to 130)	-52.9 (-57.3 to -48.5)	2.6 (2.4 to 2.8)	0.8 (0.7 to 0.9)	-69.9 (-73.3 to -66.3)
Spain	922 (888 to 957)	452 (421 to 485)	-51.0 (-54.7 to -46.7)	2.2 (2.1 to 2.3)	0.7 (0.7 to 0.8)	-66.2 (-69.1 to -63.2)
Sweden	142 (136 to 148)	109 (102 to 116)	-23.4 (-28.8 to -17.6)	1.4 (1.3 to 1.5)	0.8 (0.7 to 0.8)	-43.5 (-47.7 to -38.8)
Switzerland	91 (86 to 97)	52 (48 to 57)	-42.4 (-48.4 to -36.1)	1.3 (1.2 to 1.4)	0.5 (0.5 to 0.6)	-60.6 (-65.2 to -55.5)
United Kingdom	488 (480 to 498)	335 (327 to 343)	-31.3 (-33.5 to -29.3)	0.8 (0.8 to 0.8)	0.4 (0.4 to 0.5)	-45.4 (-47.4 to -43.5)
**Latin America and Caribbean**	**26 632 (25 719 to 27 322)**	**17 345 (16 888 to 17 799)**	**-34.9 (-37.2 to -32.3)**	**6.6 (6.4 to 6.8)**	**3.0 (2.9 to 3.1)**	**-55.1 (-56.7 to -53.5)**
**Andean Latin America**	**3 125 (2 827 to 3 407)**	**1 920 (1 730 to 2 124)**	**-38.6 (-46.4 to -29.5)**	**7.7 (7.1 to 8.3)**	**3.2 (2.9 to 3.5)**	**-58.9 (-63.7 to -53.4)**
Bolivia	836 (657 to 1 008)	429 (312 to 529)	-48.6 (-64.3 to -31.2)	11.7 (9.6 to 13.6)	3.9 (2.9 to 4.8)	-66.5 (-75.1 to -56.6)
Ecuador	706 (669 to 745)	645 (577 to 724)	-8.6 (-19.6 to 4.5)	7.3 (7.0 to 7.6)	3.9 (3.5 to 4.4)	-46.1 (-52.3 to -38.8)
Peru	1 584 (1 391 to 1 805)	846 (708 to 994)	-46.6 (-57.0 to -33.9)	6.7 (6.0 to 7.4)	2.6 (2.2 to 3.0)	-61.5 (-68.4 to -53.2)
**Caribbean**	**2 761 (2 403 to 3 106)**	**1 889 (1 681 to 2 115)**	**-31.6 (-40.4 to -20.8)**	**7.5 (6.6 to 8.4)**	**4.2 (3.7 to 4.7)**	**-44.7 (-51.7 to -35.8)**
Antigua and Barbuda	4 (4 to 5)	4 (4 to 5)	5.8 (-7.1 to 21.6)	7.0 (6.5 to 7.6)	5.0 (4.4 to 5.6)	-29.2 (-38.0 to -18.1)
The Bahamas	23 (21 to 24)	26 (23 to 29)	14.7 (0.0 to 30.3)	9.1 (8.4 to 9.7)	6.7 (6.0 to 7.5)	-25.7 (-34.7 to -16.4)
Barbados	15 (14 to 16)	11 (10 to 13)	-25.7 (-35.7 to -15.3)	5.8 (5.4 to 6.2)	3.6 (3.2 to 4.0)	-38.5 (-46.8 to -29.8)
Belize	17 (15 to 19)	26 (23 to 28)	53.0 (33.3 to 74.4)	8.3 (7.6 to 9.1)	6.6 (6.0 to 7.1)	-20.5 (-29.5 to -10.4)
Bermuda	4 (4 to 5)	2 (2 to 2)	-48.9 (-54.4 to -42.6)	7.2 (6.7 to 7.8)	2.9 (2.6 to 3.2)	-60.3 (-65.1 to -55.0)
Cuba	529 (504 to 557)	309 (275 to 351)	-41.6 (-48.5 to -33.5)	4.8 (4.6 to 5.0)	2.5 (2.2 to 2.8)	-48.9 (-55.0 to -42.0)
Dominica	5 (5 to 5)	4 (4 to 5)	-12.9 (-23.2 to -1.2)	6.6 (6.1 to 7.1)	6.2 (5.6 to 7.0)	-6.2 (-17.3 to 7.2)
Dominican Republic	333 (296 to 372)	293 (250 to 340)	-12.1 (-27.4 to 5.9)	4.5 (4.0 to 4.9)	2.9 (2.4 to 3.3)	-35.9 (-45.9 to -23.3)
Grenada	9 (8 to 9)	7 (7 to 8)	-14.8 (-24.9 to -4.8)	10.4 (9.6 to 11.2)	6.3 (5.8 to 6.9)	-39.4 (-46.3 to -31.9)
Guyana	62 (58 to 66)	55 (47 to 62)	-11.9 (-24.8 to 2.0)	8.5 (8.0 to 9.0)	7.4 (6.4 to 8.4)	-13.2 (-25.5 to -0.1)
Haiti	1 382 (1 053 to 1 713)	908 (726 to 1 131)	-34.3 (-49.2 to -13.0)	18.2 (14.2 to 21.8)	7.7 (6.2 to 9.5)	-58.0 (-66.9 to -45.5)
Jamaica	29 (26 to 32)	32 (27 to 39)	10.3 (-10.8 to 37.0)	1.2 (1.1 to 1.4)	1.1 (0.9 to 1.3)	-10.9 (-26.9 to 10.1)
Puerto Rico	122 (115 to 129)	43 (39 to 47)	-64.9 (-68.6 to -60.7)	3.3 (3.2 to 3.5)	1.1 (1.0 to 1.2)	-67.7 (-71.2 to -63.5)
Saint Lucia	11 (10 to 12)	10 (9 to 11)	-8.0 (-18.8 to 3.3)	8.3 (7.7 to 8.9)	5.3 (4.8 to 5.8)	-36.6 (-43.8 to -28.8)
Saint Vincent and the Grenadines	8 (7 to 9)	8 (7 to 8)	-4.4 (-15.4 to 8.3)	7.5 (6.9 to 8.1)	6.4 (5.8 to 7.0)	-14.5 (-24.0 to -3.2)
Suriname	30 (28 to 33)	30 (27 to 34)	0.1 (-14.1 to 17.0)	7.8 (7.1 to 8.5)	5.3 (4.7 to 6.0)	-31.8 (-41.3 to -20.4)
Trinidad and Tobago	78 (73 to 83)	47 (37 to 58)	-39.4 (-51.7 to -24.1)	6.5 (6.1 to 6.8)	3.3 (2.6 to 4.1)	-48.7 (-59.2 to -36.7)
Virgin Islands	6 (5 to 7)	5 (5 to 6)	-13.5 (-25.4 to 0.4)	6.0 (5.4 to 6.6)	4.2 (3.7 to 4.8)	-29.7 (-38.5 to -18.7)
**Central Latin America**	**10 419 (10 227 to 10 620)**	**6 969 (6 698 to 7 246)**	**-33.1 (-36.0 to -30.1)**	**6.4 (6.3 to 6.6)**	**2.7 (2.6 to 2.8)**	**-57.5 (-59.3 to -55.7)**
Colombia	2 029 (1 920 to 2 141)	1 152 (1 006 to 1 314)	-43.2 (-51.0 to -34.4)	6.0 (5.7 to 6.3)	2.3 (2.0 to 2.6)	-61.7 (-67.0 to -56.0)
Costa Rica	180 (170 to 190)	152 (138 to 169)	-15.3 (-24.5 to -3.9)	6.1 (5.8 to 6.4)	3.1 (2.8 to 3.5)	-48.6 (-53.9 to -42.1)
El Salvador	251 (225 to 281)	259 (210 to 327)	3.1 (-18.8 to 33.5)	4.7 (4.3 to 5.3)	4.1 (3.4 to 5.2)	-12.8 (-30.8 to 11.5)
Guatemala	501 (474 to 529)	774 (682 to 879)	54.5 (34.3 to 78.1)	7.6 (7.3 to 8.0)	5.0 (4.4 to 5.6)	-34.7 (-42.6 to -25.5)
Honduras	379 (315 to 437)	306 (242 to 380)	-19.3 (-37.6 to 8.9)	8.0 (6.6 to 9.2)	3.7 (2.9 to 4.5)	-54.3 (-64.5 to -39.8)
Mexico	5 532 (5 402 to 5 663)	3 303 (3 214 to 3 403)	-40.3 (-42.4 to -37.9)	6.7 (6.6 to 6.9)	2.6 (2.6 to 2.7)	-60.8 (-62.1 to -59.4)
Nicaragua	245 (216 to 278)	160 (138 to 191)	-34.7 (-45.3 to -20.3)	6.2 (5.5 to 7.0)	2.6 (2.2 to 3.1)	-58.6 (-65.2 to -49.9)
Panama	184 (171 to 196)	133 (120 to 148)	-27.7 (-36.3 to -18.6)	7.8 (7.3 to 8.3)	3.4 (3.0 to 3.8)	-56.6 (-61.6 to -51.1)
Venezuela	1 117 (1 060 to 1 182)	729 (613 to 856)	-34.7 (-45.6 to -22.3)	5.8 (5.5 to 6.1)	2.4 (2.0 to 2.8)	-59.0 (-65.7 to -51.4)
**Tropical Latin America**	**10 328 (9 677 to 10 754)**	**6 568 (6 367 to 6 750)**	**-36.4 (-39.5 to -32.6)**	**6.5 (6.1 to 6.7)**	**3.0 (2.9 to 3.1)**	**-54.0 (-56.1 to -51.8)**
Brazil	10 195 (9 551 to 10 618)	6 399 (6 202 to 6 579)	-37.2 (-40.3 to -33.4)	6.5 (6.2 to 6.8)	3.0 (2.9 to 3.1)	-54.3 (-56.4 to -52.0)
Paraguay	133 (118 to 148)	169 (135 to 209)	26.7 (-0.9 to 62.9)	3.2 (2.9 to 3.6)	2.5 (2.0 to 3.0)	-24.1 (-39.9 to -3.6)
**North Africa and Middle East**	**17 994 (14 236 to 20 964)**	**11 414 (10 562 to 12 365)**	**-36.6 (-47.1 to -18.9)**	**4.4 (3.5 to 5.0)**	**1.9 (1.7 to 2.0)**	**-56.8 (-63.7 to -45.7)**
**North Africa and Middle East**	**17 994 (14 236 to 20 964)**	**11 414 (10 562 to 12 365)**	**-36.6 (-47.1 to -18.9)**	**4.4 (3.5 to 5.0)**	**1.9 (1.7 to 2.0)**	**-56.8 (-63.7 to -45.7)**
Afghanistan	1 508 (435 to 2 259)	2 003 (1 572 to 2 503)	32.8 (-15.8 to 371.8)	11.8 (3.4 to 17.1)	5.1 (4.1 to 6.3)	-56.4 (-71.7 to 53.2)
Algeria	948 (746 to 1 144)	509 (410 to 594)	-46.3 (-57.4 to -32.5)	3.1 (2.5 to 3.7)	1.3 (1.0 to 1.5)	-59.3 (-67.2 to -49.6)
Bahrain	10 (8 to 11)	10 (8 to 11)	1.5 (-16.2 to 23.1)	2.1 (1.9 to 2.3)	0.7 (0.6 to 0.8)	-66.4 (-71.5 to -60.6)
Egypt	1 853 (1 515 to 2 176)	1 570 (1 318 to 1 863)	-15.2 (-33.2 to 9.6)	2.9 (2.4 to 3.4)	1.6 (1.4 to 1.9)	-45.2 (-56.2 to -30.0)
Iran	3 054 (2 660 to 3 383)	1 044 (1 007 to 1 110)	-65.8 (-69.5 to -60.3)	4.2 (3.7 to 4.7)	1.3 (1.3 to 1.4)	-68.5 (-71.9 to -63.5)

Location	All-age mortality counts (95% UI)			Age-standardised cause-specific mortality rates per 100,000 (95% UI)		
	1990	2017	Percentage change between 1990 and 2017	1990	2017	Percentage change between 1990 and 2017
Iraq	694 (479 to 838)	575 (500 to 671)	-17.1 (-35.3 to 16.1)	3.4 (2.5 to 4.0)	1.2 (1.0 to 1.3)	-65.4 (-72.2 to -54.0)
Jordan	126 (104 to 147)	108 (90 to 129)	-14.1 (-33.2 to 12.7)	2.7 (2.3 to 3.1)	1.0 (0.8 to 1.1)	-64.2 (-71.5 to -53.7)
Kuwait	33 (29 to 37)	24 (22 to 27)	-26.3 (-35.8 to -15.5)	1.8 (1.6 to 2.1)	0.6 (0.5 to 0.7)	-66.8 (-70.9 to -62.4)
Lebanon	102 (84 to 124)	88 (71 to 108)	-13.7 (-35.2 to 14.1)	2.3 (1.9 to 2.8)	1.0 (0.8 to 1.2)	-56.5 (-66.8 to -43.6)
Libya	139 (110 to 167)	125 (95 to 165)	-10.2 (-35.0 to 29.1)	3.0 (2.4 to 3.6)	1.8 (1.4 to 2.4)	-40.1 (-56.1 to -16.0)
Morocco	1 262 (985 to 1 527)	626 (492 to 772)	-50.4 (-62.0 to -35.8)	4.3 (3.4 to 5.1)	1.8 (1.4 to 2.2)	-58.0 (-67.6 to -46.0)
Palestine	66 (52 to 80)	41 (34 to 53)	-38.0 (-52.0 to -16.8)	2.7 (2.2 to 3.2)	0.9 (0.8 to 1.1)	-66.7 (-73.0 to -57.4)
Oman	115 (90 to 143)	97 (78 to 119)	-15.8 (-38.9 to 15.9)	5.6 (4.4 to 7.0)	2.5 (2.1 to 3.1)	-54.5 (-66.7 to -38.1)
Qatar	11 (9 to 13)	36 (28 to 45)	222.5 (139.2 to 338.0)	2.7 (2.2 to 3.2)	1.4 (1.1 to 1.7)	-47.9 (-60.8 to -31.2)
Saudi Arabia	1 308 (979 to 1 699)	1 325 (1 004 to 1 764)	1.3 (-34.8 to 61.9)	7.9 (5.9 to 10.1)	3.8 (3.0 to 4.8)	-51.9 (-68.0 to -25.5)
Sudan	2 507 (1 527 to 3 597)	1 207 (924 to 1 557)	-51.9 (-68.2 to -21.3)	8.6 (5.4 to 12.0)	2.5 (2.0 to 3.2)	-70.6 (-79.9 to -54.0)
Syria	338 (273 to 412)	152 (123 to 186)	-54.8 (-67.2 to -37.4)	2.3 (2.0 to 2.8)	0.9 (0.7 to 1.0)	-63.6 (-72.8 to -51.7)
Tunisia	257 (208 to 318)	98 (75 to 124)	-61.8 (-72.2 to -48.6)	2.7 (2.2 to 3.3)	0.9 (0.7 to 1.1)	-67.2 (-75.9 to -56.4)
Turkey	2 394 (1 918 to 2 927)	861 (754 to 977)	-64.0 (-71.7 to -53.1)	3.7 (3.0 to 4.4)	1.1 (1.0 to 1.2)	-70.1 (-76.3 to -61.2)
United Arab Emirates	55 (41 to 73)	152 (117 to 196)	177.1 (81.6 to 323.5)	3.2 (2.4 to 4.2)	1.9 (1.5 to 2.4)	-40.7 (-60.1 to -11.3)
Yemen	1 203 (495 to 1 777)	753 (564 to 1 016)	-37.4 (-59.5 to 56.2)	6.3 (2.7 to 9.2)	2.3 (1.7 to 3.1)	-63.7 (-75.4 to -12.7)
**South Asia**	**155 253 (128 706 to 177 053)**	**88 238 (81 684 to 94 567)**	**-43.2 (-50.3 to -31.3)**	**12.3 (10.4 to 13.8)**	**5.3 (4.9 to 5.7)**	**-56.6 (-61.5 to -49.2)**
**South Asia**	**155 253 (128 706 to 177 053)**	**88 238 (81 684 to 94 567)**	**-43.2 (-50.3 to -31.3)**	**12.3 (10.4 to 13.8)**	**5.3 (4.9 to 5.7)**	**-56.6 (-61.5 to -49.2)**
Bangladesh	48 958 (36 349 to 60 569)	14 029 (11 156 to 17 788)	-71.3 (-77.8 to -59.8)	32.2 (24.8 to 38.7)	9.5 (7.6 to 12.1)	-70.4 (-76.7 to -59.8)
Bhutan	49 (36 to 62)	23 (17 to 29)	-52.4 (-66.9 to -33.8)	7.6 (5.9 to 9.4)	2.6 (2.0 to 3.2)	-65.4 (-74.7 to -53.4)
India	88 624 (75 957 to 100 140)	60 199 (55 881 to 64 484)	-32.1 (-40.4 to -21.2)	9.6 (8.4 to 10.7)	4.7 (4.4 to 5.1)	-50.7 (-56.2 to -43.7)
Nepal	2 748 (1 874 to 3 695)	941 (715 to 1 143)	-65.8 (-76.0 to -45.9)	11.3 (8.2 to 14.6)	3.4 (2.7 to 4.1)	-69.6 (-78.0 to -55.3)
Pakistan	14 875 (10 210 to 20 026)	13 046 (10 228 to 15 888)	-12.3 (-36.7 to 25.8)	10.3 (7.4 to 13.4)	5.8 (4.7 to 7.0)	-43.1 (-57.5 to -23.0)
**Southeast Asia, East Asia, and Oceania**	**233 476 (216 544 to 248 298)**	**98 847 (94 676 to 102 601)**	**-57.7 (-60.2 to -54.6)**	**13.2 (12.3 to 14.0)**	**5.0 (4.8 to 5.2)**	**-62.0 (-64.2 to -59.5)**
**East Asia**	**178 957 (167 139 to 190 752)**	**67 029 (62 954 to 69 713)**	**-62.5 (-65.1 to -59.8)**	**14.6 (13.7 to 15.6)**	**5.1 (4.9 to 5.3)**	**-64.9 (-67.0 to -62.5)**
China	172 843 (161 186 to 184 525)	63 781 (59 993 to 66 409)	-63.1 (-65.6 to -60.3)	14.8 (13.8 to 15.8)	5.1 (4.9 to 5.3)	-65.3 (-67.5 to -62.9)
North Korea	1 448 (1 185 to 1 766)	1 584 (1 291 to 1 920)	9.4 (-14.7 to 44.1)	7.3 (6.0 to 8.8)	7.0 (5.7 to 8.5)	-3.5 (-24.3 to 26.1)
Taiwan (Province of China)	1 685 (1 628 to 1 743)	585 (544 to 627)	-65.3 (-67.8 to -62.5)	8.6 (8.3 to 8.9)	2.0 (1.9 to 2.2)	-76.1 (-77.7 to -74.3)
**Oceania**	**1 028 (852 to 1 219)**	**1 852 (1 500 to 2 239)**	**80.1 (46.6 to 120.4)**	**15.8 (13.2 to 18.6)**	**14.5 (11.8 to 17.4)**	**-8.3 (-25.6 to 11.2)**
American Samoa	4 (4 to 5)	4 (3 to 4)	-9.6 (-24.3 to 7.5)	9.1 (8.0 to 10.3)	7.0 (6.2 to 7.8)	-23.7 (-35.4 to -10.1)
Federated States of Micronesia	10 (8 to 12)	9 (6 to 11)	-15.7 (-43.8 to 14.1)	10.5 (8.7 to 12.9)	8.4 (5.4 to 11.0)	-20.3 (-47.4 to 6.4)
Fiji	57 (49 to 66)	64 (55 to 75)	13.4 (-8.0 to 40.0)	7.6 (6.7 to 8.7)	7.1 (6.1 to 8.3)	-6.6 (-23.8 to 14.1)
Guam	7 (7 to 8)	8 (7 to 9)	8.0 (-7.7 to 27.4)	5.5 (4.9 to 6.1)	4.7 (4.2 to 5.3)	-13.9 (-26.0 to 1.0)
Kiribati	6 (5 to 7)	6 (5 to 7)	9.6 (-15.0 to 42.1)	7.1 (6.1 to 8.1)	5.2 (4.2 to 6.3)	-26.8 (-41.9 to -7.7)
Marshall Islands	5 (4 to 6)	6 (5 to 7)	25.4 (-0.8 to 57.1)	12.1 (10.6 to 14.0)	11.0 (9.0 to 13.3)	-9.8 (-27.7 to 11.7)
Northern Mariana Islands	4 (3 to 4)	3 (3 to 4)	-12.0 (-30.6 to 11.0)	7.9 (6.6 to 9.3)	6.6 (5.7 to 7.5)	-16.5 (-32.7 to 3.3)
Papua New Guinea	797 (640 to 969)	1 541 (1 212 to 1 897)	93.4 (52.9 to 144.5)	19.3 (15.4 to 23.2)	16.4 (13.0 to 20.2)	-14.8 (-32.9 to 6.7)
Samoa	9 (8 to 11)	9 (7 to 11)	-4.0 (-26.5 to 22.7)	6.1 (5.0 to 7.3)	4.8 (3.8 to 5.9)	-20.8 (-39.4 to 0.7)
Solomon Islands	40 (32 to 49)	63 (50 to 77)	57.6 (20.7 to 102.0)	12.8 (10.2 to 16.0)	10.3 (8.2 to 12.6)	-19.5 (-38.4 to 4.4)
Tonga	6 (5 to 7)	7 (6 to 8)	14.1 (-9.5 to 46.1)	6.3 (5.5 to 7.3)	6.7 (5.5 to 8.0)	5.7 (-15.8 to 33.9)
Vanuatu	16 (12 to 21)	31 (22 to 42)	92.9 (39.9 to 165.6)	11.7 (8.6 to 15.1)	11.1 (8.0 to 15.2)	-4.7 (-30.6 to 30.3)
**Southeast Asia**	**53 491 (46 889 to 59 151)**	**29 965 (28 393 to 31 803)**	**-44.0 (-49.7 to -36.1)**	**10.5 (9.4 to 11.5)**	**4.8 (4.6 to 5.1)**	**-54.2 (-58.4 to -48.8)**
Cambodia	3 071 (2 473 to 3 652)	1 304 (1 113 to 1 537)	-57.5 (-66.4 to -44.7)	22.6 (19.1 to 26.0)	8.4 (7.3 to 9.8)	-62.8 (-69.5 to -53.4)
Indonesia	17 387 (14 900 to 19 411)	5 727 (5 285 to 6 190)	-67.1 (-70.5 to -62.1)	8.3 (7.2 to 9.2)	2.5 (2.3 to 2.7)	-69.7 (-72.6 to -65.9)
Laos	872 (658 to 1 102)	481 (371 to 591)	-44.8 (-60.2 to -28.9)	18.2 (14.3 to 22.2)	7.0 (5.5 to 8.5)	-61.8 (-71.1 to -52.1)
Malaysia	736 (645 to 829)	875 (782 to 991)	19.0 (-0.3 to 44.3)	4.6 (4.1 to 5.1)	3.0 (2.7 to 3.3)	-35.2 (-45.1 to -23.1)
Maldives	37 (28 to 46)	14 (12 to 16)	-61.7 (-69.6 to -50.1)	16.4 (13.1 to 19.4)	3.7 (3.3 to 4.1)	-77.3 (-81.3 to -71.7)
Mauritius	51 (48 to 54)	46 (42 to 51)	-9.2 (-18.8 to 1.4)	4.9 (4.6 to 5.2)	3.2 (2.9 to 3.6)	-33.8 (-40.7 to -26.1)
Myanmar	7 652 (5 861 to 9 711)	3 136 (2 632 to 3 673)	-59.0 (-69.5 to -44.0)	16.5 (12.9 to 20.6)	6.4 (5.3 to 7.5)	-61.3 (-70.9 to -48.5)
Philippines	4 977 (4 466 to 5 502)	5 245 (4 635 to 5 970)	5.4 (-10.3 to 25.1)	7.4 (6.8 to 8.0)	5.3 (4.7 to 6.0)	-28.6 (-38.0 to -17.0)
Sri Lanka	1 249 (1 136 to 1 353)	1 037 (857 to 1 237)	-17.0 (-32.2 to 1.0)	8.3 (7.7 to 8.9)	4.7 (3.9 to 5.5)	-43.7 (-53.8 to -31.6)
Seychelles	10 (10 to 11)	12 (11 to 14)	18.2 (5.6 to 33.6)	15.9 (14.6 to 17.1)	11.8 (10.8 to 12.8)	-25.7 (-33.7 to -16.6)
Thailand	6 049 (5 152 to 6 903)	5 045 (4 481 to 5 651)	-16.6 (-30.5 to 2.3)	10.7 (9.2 to 12.1)	7.0 (6.2 to 7.7)	-35.0 (-44.7 to -22.2)
Timor-Leste	122 (85 to 159)	70 (40 to 88)	-42.2 (-66.5 to -15.9)	12.8 (9.7 to 16.0)	5.3 (3.2 to 6.4)	-58.6 (-74.8 to -44.2)
Vietnam	11 206 (9 147 to 13 382)	6 932 (5 983 to 7 854)	-38.1 (-50.2 to -22.2)	15.1 (12.7 to 17.7)	7.7 (6.7 to 8.8)	-48.7 (-57.9 to -37.6)
**Sub-Saharan Africa**	**43 056 (33 992 to 51 701)**	**41 753 (37 181 to 46 533)**	**-3.0 (-19.9 to 25.1)**	**7.2 (6.0 to 8.4)**	**3.9 (3.6 to 4.3)**	**-45.6 (-53.6 to -33.7)**
**Central sub-Saharan Africa**	**9 065 (6 114 to 12 063)**	**8 746 (6 964 to 11 080)**	**-3.5 (-29.5 to 40.6)**	**11.7 (8.4 to 15.2)**	**6.0 (4.9 to 7.3)**	**-48.8 (-61.1 to -29.7)**
Angola	2 746 (1 488 to 4 076)	2 005 (1 517 to 2 569)	-27.0 (-52.8 to 27.7)	18.4 (10.8 to 26.5)	5.9 (4.7 to 7.3)	-68.1 (-77.9 to -46.7)
Central African Republic	424 (227 to 595)	495 (352 to 677)	16.9 (-20.3 to 124.4)	12.3 (7.4 to 16.4)	9.7 (7.1 to 12.8)	-20.6 (-43.6 to 38.3)
Congo (Brazzaville)	335 (243 to 447)	247 (185 to 317)	-26.2 (-46.9 to 4.0)	11.3 (8.8 to 14.5)	4.9 (3.7 to 6.1)	-56.9 (-67.8 to -42.9)
DR Congo	5 380 (3 474 to 7 481)	5 878 (4 265 to 8 010)	9.3 (-28.0 to 72.7)	9.9 (6.7 to 13.1)	5.9 (4.6 to 7.7)	-39.7 (-57.4 to -12.0)
Equatorial Guinea	86 (59 to 116)	50 (33 to 74)	-42.0 (-63.9 to -8.1)	14.6 (10.7 to 19.0)	3.6 (2.4 to 5.3)	-75.5 (-84.3 to -63.1)

Location	All-age mortality counts (95% UI)			Age-standardised cause-specific mortality rates per 100,000 (95% UI)		
	1990	2017	Percentage change between 1990 and 2017	1990	2017	Percentage change between 1990 and 2017
Gabon	95 (70 to 128)	71 (55 to 90)	-25.4 (-46.6 to 6.9)	8.5 (6.6 to 11.2)	4.2 (3.3 to 5.2)	-50.9 (-63.5 to -31.8)
**Eastern sub-Saharan Africa**	**15 849 (11 559 to 19 630)**	**12 605 (11 432 to 14 030)**	**-20.5 (-35.5 to 8.6)**	**7.1 (5.5 to 8.4)**	**3.4 (3.2 to 3.7)**	**-51.6 (-59.4 to -38.0)**
Burundi	506 (379 to 646)	443 (347 to 563)	-12.5 (-36.0 to 22.3)	8.3 (6.8 to 9.9)	4.2 (3.5 to 5.2)	-48.9 (-59.5 to -34.8)
Comoros	32 (25 to 39)	18 (15 to 22)	-41.7 (-55.0 to -23.8)	6.5 (5.3 to 7.7)	2.9 (2.5 to 3.5)	-55.0 (-63.9 to -43.3)
Djibouti	33 (19 to 44)	28 (21 to 40)	-13.3 (-41.2 to 48.5)	6.2 (4.1 to 8.0)	3.1 (2.3 to 4.2)	-50.9 (-65.0 to -25.1)
Eritrea	269 (133 to 384)	241 (189 to 303)	-10.1 (-40.1 to 80.4)	8.7 (5.3 to 11.4)	4.6 (3.8 to 5.6)	-47.3 (-61.8 to -12.0)
Ethiopia	5 522 (3 709 to 7 142)	2 991 (2 571 to 3 513)	-45.8 (-59.7 to -17.0)	9.2 (6.7 to 11.3)	3.1 (2.8 to 3.5)	-66.1 (-73.4 to -53.2)
Kenya	959 (769 to 1 130)	1 106 (1 009 to 1 217)	15.4 (-1.9 to 41.7)	4.3 (3.4 to 5.0)	2.9 (2.7 to 3.1)	-32.5 (-40.9 to -19.8)
Madagascar	1 008 (809 to 1 224)	833 (649 to 1 069)	-17.3 (-37.3 to 12.5)	7.1 (6.0 to 8.4)	3.4 (2.8 to 4.2)	-52.3 (-61.9 to -39.9)
Malawi	922 (341 to 1 378)	576 (463 to 719)	-37.5 (-59.7 to 71.6)	7.2 (3.1 to 10.3)	3.5 (2.9 to 4.1)	-52.0 (-67.2 to 15.6)
Mozambique	1 496 (1 083 to 1 950)	1 168 (960 to 1 417)	-21.9 (-43.7 to 11.0)	8.6 (6.8 to 10.7)	4.5 (3.8 to 5.3)	-48.4 (-60.1 to -31.2)
Rwanda	644 (487 to 808)	357 (289 to 450)	-44.6 (-59.1 to -19.8)	8.0 (6.6 to 9.4)	3.1 (2.6 to 3.7)	-61.3 (-69.3 to -49.9)
Somalia	653 (202 to 1 081)	728 (497 to 1 042)	11.4 (-37.5 to 252.5)	7.7 (3.2 to 12.0)	4.5 (3.3 to 6.1)	-41.7 (-64.5 to 35.5)
South Sudan	456 (175 to 763)	579 (407 to 783)	26.9 (-20.0 to 182.9)	7.3 (3.4 to 11.6)	5.5 (4.0 to 7.0)	-25.3 (-48.4 to 38.6)
Tanzania	1 349 (665 to 1 826)	1 819 (1 443 to 2 306)	34.9 (-7.2 to 188.2)	4.7 (2.7 to 5.9)	3.3 (2.8 to 3.9)	-29.5 (-46.8 to 19.9)
Uganda	1 174 (806 to 1 623)	1 084 (862 to 1 369)	-7.7 (-34.1 to 37.1)	5.2 (3.9 to 6.7)	2.9 (2.4 to 3.4)	-44.6 (-57.6 to -25.4)
Zambia	821 (487 to 1 125)	626 (496 to 791)	-23.8 (-47.7 to 34.9)	8.4 (5.5 to 10.8)	3.8 (3.2 to 4.5)	-55.2 (-66.5 to -29.1)
**Southern sub-Saharan Africa**	**2 558 (2 325 to 2 799)**	**2 450 (2 280 to 2 629)**	**-4.2 (-14.6 to 8.4)**	**5.2 (4.8 to 5.7)**	**3.3 (3.1 to 3.5)**	**-36.5 (-43.0 to -29.0)**
Botswana	50 (39 to 61)	45 (39 to 54)	-8.9 (-27.9 to 18.0)	4.5 (3.5 to 5.4)	2.2 (1.9 to 2.6)	-50.5 (-60.7 to -37.0)
Lesotho	87 (71 to 103)	112 (91 to 136)	28.9 (-0.0 to 66.3)	5.5 (4.5 to 6.4)	6.2 (5.1 to 7.4)	12.5 (-11.7 to 44.3)
Namibia	69 (56 to 82)	68 (55 to 84)	-1.7 (-23.7 to 27.0)	5.6 (4.6 to 6.6)	3.2 (2.6 to 3.9)	-43.0 (-55.0 to -27.0)
South Africa	1 827 (1 667 to 2 003)	1 439 (1 340 to 1 560)	-21.2 (-29.4 to -12.4)	5.2 (4.7 to 5.7)	2.6 (2.5 to 2.9)	-49.0 (-54.1 to -43.7)
Swaziland	41 (34 to 50)	55 (43 to 69)	33.7 (-1.5 to 81.4)	5.9 (5.0 to 7.0)	5.4 (4.3 to 6.7)	-8.6 (-31.2 to 20.5)
Zimbabwe	484 (400 to 563)	730 (616 to 865)	51.0 (18.6 to 92.5)	5.5 (4.8 to 6.3)	5.9 (5.0 to 6.9)	6.2 (-13.7 to 30.6)
**Western sub-Saharan Africa**	**15 585 (12 484 to 18 624)**	**17 952 (15 141 to 21 344)**	**15.2 (-7.3 to 51.9)**	**6.5 (5.5 to 7.6)**	**3.8 (3.4 to 4.5)**	**-40.7 (-50.1 to -26.9)**
Benin	442 (330 to 564)	420 (319 to 559)	-4.9 (-32.3 to 31.6)	7.2 (5.8 to 8.8)	3.7 (3.0 to 4.6)	-48.4 (-59.4 to -33.9)
Burkina Faso	691 (525 to 870)	1 210 (895 to 1 604)	75.1 (22.7 to 156.1)	6.0 (4.9 to 7.2)	5.1 (4.1 to 6.2)	-16.4 (-35.6 to 12.8)
Cameroon	671 (518 to 863)	882 (674 to 1 112)	31.4 (-5.0 to 88.4)	6.0 (5.0 to 7.2)	3.6 (2.9 to 4.3)	-40.4 (-54.4 to -21.9)
Cape Verde	17 (15 to 20)	27 (23 to 32)	55.6 (25.4 to 92.0)	5.0 (4.3 to 5.7)	5.1 (4.4 to 6.0)	2.7 (-15.2 to 23.9)
Chad	512 (352 to 705)	980 (738 to 1 301)	91.2 (33.9 to 178.5)	6.6 (4.8 to 8.5)	5.4 (4.3 to 6.7)	-17.5 (-36.4 to 6.8)
Cote d'Ivoire	876 (663 to 1 128)	1 042 (836 to 1 299)	19.0 (-12.7 to 66.8)	6.5 (5.2 to 7.8)	4.4 (3.7 to 5.2)	-32.2 (-47.5 to -13.1)
The Gambia	71 (51 to 94)	68 (50 to 90)	-3.8 (-31.5 to 33.6)	6.1 (4.7 to 7.7)	3.6 (2.9 to 4.5)	-40.7 (-54.3 to -22.3)
Ghana	567 (463 to 705)	790 (650 to 954)	39.4 (6.5 to 82.6)	4.0 (3.4 to 4.7)	3.2 (2.7 to 3.7)	-21.0 (-35.9 to -2.2)
Guinea	805 (553 to 1 102)	598 (468 to 754)	-25.8 (-49.2 to 12.9)	9.4 (6.9 to 12.3)	5.0 (4.1 to 6.0)	-47.0 (-60.7 to -25.6)
Guinea-Bissau	131 (88 to 188)	91 (65 to 124)	-30.3 (-50.6 to 2.9)	11.2 (8.1 to 15.2)	5.4 (4.1 to 6.9)	-52.3 (-64.2 to -36.3)
Liberia	226 (163 to 294)	132 (97 to 185)	-41.4 (-59.0 to -10.3)	8.5 (6.6 to 10.7)	3.2 (2.5 to 4.0)	-62.8 (-72.0 to -48.1)
Mali	1 175 (830 to 1 642)	1 523 (1 087 to 2 126)	29.6 (-11.8 to 96.6)	9.7 (7.4 to 12.7)	5.7 (4.4 to 7.5)	-40.9 (-56.2 to -17.3)
Mauritania	98 (79 to 122)	87 (68 to 116)	-11.3 (-34.1 to 22.9)	4.8 (4.0 to 5.8)	2.5 (2.0 to 3.1)	-48.0 (-58.7 to -33.9)
Niger	1 368 (893 to 1 950)	1 203 (862 to 1 669)	-12.0 (-42.6 to 39.0)	11.3 (8.0 to 15.3)	4.6 (3.5 to 5.9)	-59.3 (-70.8 to -42.5)
Nigeria	6 520 (4 955 to 8 445)	7 812 (5 733 to 10 526)	19.8 (-16.7 to 74.9)	5.8 (4.7 to 7.3)	3.3 (2.5 to 4.3)	-43.7 (-58.5 to -24.3)
Sao Tome and Principe	6 (5 to 7)	4 (3 to 5)	-36.4 (-53.1 to -12.1)	4.1 (3.4 to 4.8)	2.2 (1.9 to 2.8)	-44.6 (-57.8 to -26.1)
Senegal	677 (497 to 908)	503 (369 to 675)	-25.7 (-45.5 to 7.1)	7.4 (5.7 to 9.4)	3.8 (2.9 to 4.8)	-48.4 (-59.1 to -32.4)
Sierra Leone	484 (355 to 652)	377 (283 to 490)	-22.2 (-46.2 to 14.2)	9.6 (7.4 to 12.6)	4.8 (3.8 to 5.8)	-50.6 (-63.6 to -32.1)
Togo	248 (190 to 316)	203 (154 to 266)	-18.0 (-40.6 to 16.9)	5.9 (4.9 to 7.1)	3.1 (2.5 to 3.9)	-46.5 (-58.5 to -29.7)
