# Supplementary material for: The burden of unintentional drowning: global, regional and national estimates of mortality from the Global Burden of Disease 2017 Study
Source: Inj Prev. 2020 Feb 20;26(Suppl 1):i83–95. doi: 10.1136/injuryprev-2019-043484 (PMC7571364; doi:10.1136/injuryprev-2019-043484)
Supplement: Supplementary data [file injuryprev-2019-043484supp004.pdf]

Table 2: Years of life lost in and percentage change between 1990 and 2017 for drowning

Location	All-age years of life lost (95% UI)			Age-standardised years of life lost rates per 100,000 (95% UI)		
	1990	2017	Percentage change between 1990 and 2017	1990	2017	Percentage change between 1990 and 2017
**Global**	**37 925 314 (33 968 621 to 41 237 399)**	**16 563 278 (15 784 185 to 17 349 952)**	**-56.3 (-59.7 to -51.2)**	**632.3 (568.8 to 685.6)**	**228.3 (217.2 to 239.7)**	**-63.9 (-66.6 to -59.8)**
**Low SDI**	**9 116 824 (7 095 421 to 10 737 860)**	**4 574 366 (4 138 617 to 5 062 619)**	**-49.8 (-57.8 to -34.6)**	**892.6 (702.4 to 1 042.9)**	**302.7 (276.1 to 332.0)**	**-66.1 (-71.2 to -56.6)**
**Low-middle SDI**	**7 974 483 (6 656 122 to 9 044 043)**	**4 615 451 (4 270 877 to 5 000 768)**	**-42.1 (-49.4 to -31.0)**	**604.6 (511.6 to 679.9)**	**255.5 (237.0 to 276.2)**	**-57.8 (-62.7 to -50.4)**
**Middle SDI**	**13 664 873 (12 624 717 to 14 606 984)**	**4 564 996 (4 377 605 to 4 766 266)**	**-66.6 (-68.7 to -63.9)**	**776.9 (718.8 to 828.9)**	**238.7 (228.5 to 250.0)**	**-69.3 (-71.2 to -66.8)**
**High-middle SDI**	**5 714 672 (5 436 599 to 6 028 701)**	**2 099 435 (2 036 041 to 2 170 556)**	**-63.3 (-65.1 to -61.2)**	**507.6 (482.8 to 535.8)**	**171.5 (165.8 to 178.5)**	**-66.2 (-68.0 to -64.2)**
**High SDI**	**1 220 565 (1 201 599 to 1 241 728)**	**642 861 (630 112 to 656 130)**	**-47.3 (-48.6 to -46.0)**	**141.7 (139.2 to 144.6)**	**59.3 (58.0 to 60.7)**	**-58.2 (-59.4 to -56.9)**
**Central Europe, Eastern Europe, and Central Asia**	**2 057 195 (2 010 088 to 2 111 877)**	**928 902 (901 524 to 959 082)**	**-54.8 (-56.2 to -53.6)**	**510.8 (498.9 to 524.4)**	**237.4 (229.8 to 245.9)**	**-53.5 (-55.1 to -52.0)**
**Central Asia**	**522 218 (498 166 to 551 667)**	**253 637 (237 343 to 271 637)**	**-51.4 (-55.2 to -47.5)**	**653.0 (626.2 to 685.5)**	**272.9 (255.4 to 292.1)**	**-58.2 (-61.3 to -55.0)**
Armenia	15 955 (14 764 to 17 248)	2 359 (2 159 to 2 576)	-85.2 (-87.0 to -83.2)	445.0 (412.3 to 479.7)	86.7 (78.3 to 95.6)	-80.5 (-83.0 to -77.8)
Azerbaijan	37 911 (34 302 to 42 654)	17 662 (14 999 to 20 865)	-53.4 (-61.1 to -44.9)	467.4 (423.9 to 523.5)	180.1 (152.1 to 215.9)	-61.5 (-67.9 to -54.5)
Georgia	23 997 (22 284 to 25 832)	6 206 (5 700 to 6 779)	-74.1 (-76.9 to -70.9)	447.5 (414.5 to 483.3)	177.4 (161.0 to 195.5)	-60.4 (-64.9 to -55.0)
Kazakhstan	130 680 (120 305 to 140 337)	52 358 (46 533 to 59 241)	-59.9 (-65.1 to -54.1)	731.7 (676.3 to 782.9)	289.5 (257.3 to 327.0)	-60.4 (-65.4 to -54.8)
Kyrgyzstan	42 646 (38 788 to 47 279)	17 225 (15 279 to 19 444)	-59.6 (-65.5 to -52.7)	843.1 (776.6 to 923.4)	255.0 (228.6 to 285.7)	-69.8 (-73.7 to -65.1)
Mongolia	10 662 (8 531 to 13 179)	8 383 (7 070 to 9 979)	-21.4 (-37.4 to -0.7)	395.4 (325.9 to 479.9)	247.4 (209.8 to 292.9)	-37.4 (-49.2 to -22.7)
Tajikistan	51 557 (45 716 to 59 302)	47 603 (41 179 to 55 991)	-7.7 (-24.0 to 12.3)	734.2 (665.7 to 823.7)	452.8 (396.1 to 524.7)	-38.3 (-48.1 to -26.4)
Turkmenistan	38 721 (32 863 to 45 275)	9 569 (8 227 to 11 458)	-75.3 (-80.5 to -68.1)	782.8 (679.7 to 898.3)	183.8 (158.3 to 219.9)	-76.5 (-81.2 to -70.3)
Uzbekistan	170 090 (151 467 to 191 527)	92 270 (80 781 to 105 897)	-45.8 (-55.1 to -35.1)	631.5 (571.6 to 699.7)	275.9 (241.4 to 316.0)	-56.3 (-63.6 to -48.6)
**Central Europe**	**291 600 (284 192 to 299 313)**	**101 353 (97 032 to 106 296)**	**-65.2 (-66.8 to -63.7)**	**249.4 (242.6 to 256.5)**	**90.8 (86.8 to 95.5)**	**-63.6 (-65.3 to -61.7)**
Albania	10 141 (8 868 to 11 347)	2 046 (1 657 to 2 486)	-79.8 (-84.1 to -74.6)	268.5 (235.8 to 299.3)	86.2 (69.8 to 104.4)	-67.9 (-74.5 to -59.9)
Bosnia and Herzegovina	2 605 (2 108 to 3 217)	1 414 (1 254 to 1 586)	-45.7 (-58.8 to -31.1)	58.1 (46.9 to 72.6)	45.0 (39.5 to 51.3)	-22.5 (-40.1 to 0.0)
Bulgaria	17 540 (16 628 to 18 462)	5 815 (5 310 to 6 378)	-66.8 (-70.1 to -63.5)	224.9 (211.6 to 238.4)	94.9 (85.4 to 105.4)	-57.8 (-62.5 to -52.8)
Croatia	6 796 (6 432 to 7 200)	2 865 (2 642 to 3 118)	-57.8 (-61.8 to -53.4)	143.5 (135.0 to 153.1)	65.7 (60.2 to 72.5)	-54.2 (-58.6 to -48.5)
Czech Republic	15 666 (14 888 to 16 516)	6 862 (6 328 to 7 457)	-56.2 (-60.2 to -52.0)	166.7 (157.0 to 177.2)	62.5 (57.0 to 68.7)	-62.5 (-66.4 to -58.4)
Hungary	17 190 (16 381 to 18 118)	5 193 (4 782 to 5 611)	-69.8 (-72.6 to -66.9)	176.0 (166.7 to 185.9)	57.3 (52.2 to 62.4)	-67.5 (-70.9 to -63.9)
Macedonia	1 714 (1 519 to 1 923)	1 139 (1 018 to 1 266)	-33.5 (-43.1 to -21.8)	87.5 (77.4 to 98.9)	58.2 (51.5 to 66.2)	-33.5 (-44.1 to -21.1)
Montenegro	514 (452 to 587)	251 (221 to 282)	-51.2 (-59.6 to -41.9)	84.0 (73.7 to 96.6)	41.8 (36.7 to 47.0)	-50.3 (-59.3 to -40.4)
Poland	98 848 (94 549 to 103 271)	36 667 (33 697 to 39 912)	-62.9 (-66.3 to -59.2)	264.2 (252.1 to 276.9)	92.8 (85.1 to 100.9)	-64.9 (-68.1 to -61.1)
Romania	93 767 (90 094 to 97 868)	29 138 (27 002 to 31 526)	-68.9 (-71.4 to -66.2)	426.4 (408.6 to 447.0)	159.9 (147.1 to 173.4)	-62.5 (-65.7 to -59.0)
Serbia	14 485 (12 962 to 16 213)	4 347 (3 946 to 4 808)	-70.0 (-74.2 to -65.2)	171.0 (151.7 to 193.9)	49.7 (44.8 to 55.6)	-70.9 (-75.4 to -65.7)
Slovakia	10 308 (9 416 to 11 242)	4 880 (4 374 to 5 465)	-52.7 (-58.7 to -45.2)	197.9 (180.2 to 216.7)	89.7 (80.1 to 101.0)	-54.7 (-60.8 to -47.1)
Slovenia	2 024 (1 898 to 2 157)	735 (670 to 801)	-63.7 (-67.6 to -59.6)	105.6 (98.5 to 113.8)	35.0 (31.7 to 38.4)	-66.9 (-70.7 to -62.8)
**Eastern Europe**	**1 243 376 (1 202 518 to 1 282 045)**	**573 912 (555 972 to 592 730)**	**-53.8 (-55.3 to -52.4)**	**577.9 (560.2 to 595.9)**	**279.4 (270.3 to 288.4)**	**-51.6 (-53.3 to -50.1)**
Belarus	70 059 (66 366 to 74 245)	26 933 (24 221 to 29 617)	-61.6 (-65.8 to -57.2)	711.6 (669.1 to 757.8)	285.8 (254.6 to 314.6)	-59.8 (-64.4 to -54.8)
Estonia	11 338 (10 780 to 11 917)	1 911 (1 666 to 2 183)	-83.1 (-85.5 to -80.5)	779.3 (738.4 to 824.2)	150.8 (130.8 to 172.0)	-80.6 (-83.4 to -77.6)
Latvia	23 106 (22 101 to 24 177)	5 747 (5 036 to 6 570)	-75.1 (-78.4 to -71.4)	911.7 (869.4 to 955.7)	300.8 (264.5 to 343.3)	-67.0 (-71.0 to -62.1)
Lithuania	28 482 (27 303 to 29 694)	8 269 (7 591 to 9 038)	-71.0 (-73.6 to -68.0)	787.4 (753.3 to 822.2)	282.1 (259.2 to 306.7)	-64.2 (-67.6 to -60.6)
Moldova	31 679 (29 587 to 33 702)	9 327 (8 644 to 10 017)	-70.6 (-73.2 to -67.7)	718.9 (671.6 to 766.0)	264.2 (241.6 to 289.4)	-63.3 (-67.0 to -59.1)
Russian	742 121 (705 841 to 769 630)	375 829 (365 281 to 386 322)	-49.4 (-50.8 to -47.6)	511.1 (487.6 to 529.9)	260.6 (252.4 to 268.2)	-49.0 (-50.4 to -47.6)
Ukraine	336 593 (321 363 to 353 594)	145 895 (135 950 to 157 031)	-56.7 (-60.4 to -52.6)	693.8 (657.9 to 732.4)	345.1 (320.4 to 373.3)	-50.3 (-54.9 to -45.2)
**High-income**	**987 659 (970 445 to 1 006 473)**	**584 612 (571 858 to 597 369)**	**-40.8 (-42.4 to -39.1)**	**121.8 (119.3 to 124.5)**	**57.3 (55.9 to 58.7)**	**-53.0 (-54.5 to -51.5)**
**Australasia**	**21 630 (20 487 to 23 017)**	**12 923 (11 665 to 14 263)**	**-40.3 (-47.0 to -32.9)**	**118.6 (111.4 to 127.3)**	**52.1 (46.7 to 57.9)**	**-56.1 (-61.5 to -50.3)**
Australia	17 328 (16 199 to 18 590)	10 253 (9 020 to 11 565)	-40.8 (-48.9 to -31.9)	115.4 (107.0 to 125.3)	49.0 (42.5 to 56.2)	-57.5 (-63.8 to -50.7)
New Zealand	4 302 (4 036 to 4 598)	2 670 (2 471 to 2 872)	-37.9 (-43.9 to -31.8)	134.3 (125.5 to 144.2)	69.1 (63.6 to 74.7)	-48.5 (-53.7 to -42.8)
**High-income Asia-Pacific**	**289 163 (278 597 to 301 060)**	**153 366 (147 752 to 158 976)**	**-47.0 (-49.5 to -44.0)**	**188.9 (180.6 to 198.3)**	**61.2 (58.6 to 63.7)**	**-67.6 (-69.5 to -65.5)**
Brunei	1 272 (1 111 to 1 432)	1 017 (892 to 1 154)	-20.0 (-33.2 to -4.6)	455.6 (404.4 to 507.5)	232.4 (204.7 to 261.7)	-49.0 (-57.1 to -39.6)
Japan	124 518 (122 138 to 127 103)	125 718 (121 517 to 130 251)	1.0 (-2.7 to 4.8)	112.6 (110.0 to 115.6)	65.1 (62.5 to 67.4)	-42.2 (-44.7 to -39.8)
South Korea	160 268 (149 826 to 171 426)	25 695 (23 163 to 28 369)	-84.0 (-85.9 to -81.9)	357.3 (331.6 to 385.3)	49.0 (44.2 to 54.4)	-86.3 (-87.9 to -84.5)
Singapore	3 104 (2 884 to 3 347)	935 (853 to 1 030)	-69.9 (-72.9 to -66.4)	110.7 (102.6 to 119.4)	18.1 (16.4 to 20.1)	-83.6 (-85.4 to -81.6)
**High-income North America**	**287 956 (282 387 to 294 561)**	**216 737 (208 568 to 225 556)**	**-24.7 (-28.2 to -21.1)**	**113.3 (110.9 to 116.1)**	**69.8 (67.0 to 72.6)**	**-38.4 (-41.4 to -35.5)**
Canada	22 996 (21 770 to 24 256)	14 623 (13 308 to 15 977)	-36.4 (-42.6 to -29.5)	93.1 (87.5 to 99.0)	47.9 (43.0 to 53.2)	-48.5 (-54.2 to -42.0)
Greenland	500 (438 to 599)	170 (152 to 190)	-65.9 (-73.1 to -59.0)	862.4 (755.4 to 1 030.8)	308.3 (270.5 to 349.2)	-64.2 (-72.2 to -56.3)
USA	264 453 (259 130 to 270 961)	201 940 (194 061 to 210 503)	-23.6 (-27.4 to -19.8)	115.2 (112.7 to 118.2)	72.0 (69.1 to 75.1)	-37.5 (-40.7 to -34.3)
**Southern Latin America**	**110 806 (105 895 to 115 566)**	**62 163 (56 962 to 68 300)**	**-43.9 (-49.0 to -37.7)**	**220.3 (210.5 to 229.5)**	**99.3 (90.8 to 109.2)**	**-54.9 (-59.0 to -50.0)**
Argentina	73 258 (68 626 to 77 502)	39 405 (34 908 to 44 619)	-46.2 (-52.8 to -38.4)	218.8 (205.3 to 231.5)	93.5 (83.1 to 105.4)	-57.2 (-62.6 to -51.0)
Chile	28 331 (26 581 to 30 190)	18 260 (15 941 to 20 811)	-35.5 (-44.8 to -24.7)	208.0 (195.4 to 221.2)	103.4 (90.6 to 117.1)	-50.3 (-57.2 to -42.3)
Uruguay	9 213 (8 725 to 9 739)	4 496 (3 930 to 5 107)	-51.2 (-57.6 to -44.1)	304.4 (288.1 to 322.5)	139.1 (121.8 to 157.7)	-54.3 (-60.4 to -47.7)
**Western Europe**	**278 105 (271 337 to 284 561)**	**139 424 (133 921 to 144 282)**	**-49.9 (-52.2 to -47.8)**	**80.7 (78.2 to 83.2)**	**33.2 (31.8 to 34.6)**	**-58.8 (-61.1 to -56.8)**
Andorra	28 (21 to 37)	18 (15 to 22)	-35.9 (-51.7 to -16.4)	61.9 (46.6 to 83.1)	25.3 (19.9 to 31.6)	-59.1 (-71.0 to -43.1)
Austria	5 594 (5 178 to 6 085)	2 253 (2 066 to 2 452)	-59.7 (-64.4 to -54.7)	83.1 (75.1 to 93.0)	26.7 (24.0 to 29.5)	-67.9 (-72.4 to -63.1)
Belgium	6 354 (5 940 to 6 820)	3 353 (3 081 to 3 648)	-47.2 (-52.4 to -41.2)	72.1 (65.9 to 79.0)	30.2 (27.6 to 33.3)	-58.1 (-63.1 to -52.1)
Cyprus	1 048 (921 to 1 170)	837 (742 to 934)	-20.2 (-32.1 to -5.1)	134.7 (118.5 to 151.0)	59.9 (53.2 to 67.4)	-55.5 (-62.2 to -46.8)

Location	All-age years of life lost (95% UI)			Age-standardised years of life lost rates per 100,000 (95% UI)		
	1990	2017	Percentage change between 1990 and 2017	1990	2017	Percentage change between 1990 and 2017
Denmark	2 157 (1 996 to 2 355)	1 248 (1 139 to 1 365)	-42.1 (-48.7 to -34.8)	49.1 (44.2 to 55.5)	22.4 (20.1 to 24.8)	-54.4 (-61.0 to -46.9)
Finland	7 432 (7 022 to 7 868)	3 819 (3 517 to 4 168)	-48.6 (-53.6 to -43.1)	146.7 (137.3 to 156.9)	64.0 (58.6 to 70.0)	-56.3 (-61.2 to -50.9)
France	51 690 (48 899 to 54 732)	35 646 (33 096 to 38 333)	-31.0 (-37.1 to -24.6)	95.8 (89.2 to 102.7)	54.9 (50.2 to 59.9)	-42.7 (-48.9 to -36.3)
Germany	51 563 (48 081 to 55 201)	18 441 (16 518 to 20 653)	-64.2 (-68.7 to -59.3)	82.2 (74.8 to 89.8)	24.2 (21.5 to 27.4)	-70.5 (-74.8 to -66.0)
Greece	11 022 (10 502 to 11 571)	9 683 (8 981 to 10 420)	-12.1 (-19.8 to -4.0)	104.5 (98.7 to 111.1)	80.0 (73.7 to 86.7)	-23.5 (-30.4 to -15.2)
Iceland	209 (189 to 231)	146 (133 to 159)	-30.0 (-38.5 to -20.7)	84.5 (76.0 to 93.9)	45.7 (40.8 to 50.5)	-45.9 (-53.4 to -37.6)
Ireland	3 670 (3 432 to 3 918)	1 873 (1 656 to 2 091)	-49.0 (-55.5 to -42.8)	103.6 (96.6 to 110.6)	38.8 (33.9 to 43.7)	-62.6 (-67.6 to -57.6)
Israel	4 071 (3 756 to 4 435)	2 552 (2 322 to 2 813)	-37.3 (-44.9 to -28.7)	80.0 (74.1 to 87.1)	29.1 (26.5 to 32.2)	-63.6 (-68.0 to -58.5)
Italy	31 506 (30 031 to 33 129)	15 026 (13 712 to 16 271)	-52.3 (-57.1 to -47.4)	58.7 (55.6 to 62.2)	27.9 (25.3 to 30.7)	-52.4 (-57.7 to -46.8)
Luxembourg	199 (185 to 215)	123 (108 to 139)	-38.1 (-46.8 to -28.6)	55.8 (51.0 to 61.1)	20.9 (18.4 to 23.6)	-62.5 (-68.3 to -55.9)
Malta	222 (199 to 246)	175 (162 to 190)	-21.3 (-30.3 to -10.7)	64.6 (57.0 to 72.1)	43.3 (39.4 to 47.9)	-33.0 (-41.7 to -22.8)
Netherlands	7 079 (6 379 to 7 942)	3 559 (3 295 to 3 870)	-49.7 (-56.6 to -42.4)	60.4 (53.1 to 69.4)	22.6 (20.4 to 25.1)	-62.5 (-68.7 to -55.4)
Norway	4 277 (4 141 to 4 403)	2 061 (1 962 to 2 146)	-51.8 (-54.8 to -49.1)	110.0 (105.9 to 113.8)	38.4 (36.4 to 40.1)	-65.1 (-67.5 to -63.1)
Portugal	13 532 (12 603 to 14 490)	3 691 (3 331 to 4 043)	-72.7 (-75.9 to -69.4)	154.6 (142.1 to 168.7)	34.8 (30.9 to 38.8)	-77.5 (-80.7 to -73.9)
Spain	42 812 (40 669 to 44 902)	15 301 (14 129 to 16 541)	-64.3 (-67.7 to -61.0)	114.6 (107.8 to 121.6)	33.8 (30.8 to 36.8)	-70.6 (-73.6 to -67.3)
Sweden	5 538 (5 274 to 5 824)	3 561 (3 325 to 3 815)	-35.7 (-40.7 to -29.9)	66.7 (62.7 to 71.2)	34.1 (31.4 to 36.8)	-48.8 (-53.4 to -43.7)
Switzerland	4 483 (4 139 to 4 882)	1 974 (1 797 to 2 161)	-56.0 (-61.3 to -50.1)	76.0 (68.5 to 85.1)	26.3 (23.3 to 29.4)	-65.3 (-70.5 to -59.8)
United Kingdom	23 351 (22 857 to 23 993)	13 939 (13 552 to 14 274)	-40.3 (-42.6 to -38.2)	45.0 (43.9 to 46.4)	22.5 (21.8 to 23.1)	-50.0 (-52.2 to -48.0)
**Latin America and Caribbean**	**1 742 425 (1 671 038 to 1 796 163)**	**946 783 (917 588 to 975 336)**	**-45.7 (-47.9 to -43.2)**	**395.4 (380.7 to 406.7)**	**164.0 (158.8 to 169.2)**	**-58.5 (-60.1 to -56.7)**
**Andean Latin America**	**215 155 (190 431 to 238 044)**	**105 266 (94 272 to 117 702)**	**-51.1 (-58.2 to -42.5)**	**469.6 (423.2 to 513.0)**	**168.1 (150.9 to 187.8)**	**-64.2 (-69.0 to -58.4)**
Bolivia	59 537 (45 653 to 73 691)	23 938 (16 087 to 30 223)	-59.8 (-75.0 to -43.2)	713.7 (563.0 to 857.3)	197.7 (137.2 to 245.6)	-72.3 (-81.9 to -62.2)
Ecuador	44 165 (41 475 to 47 216)	36 302 (32 145 to 41 197)	-17.8 (-28.9 to -4.9)	402.0 (379.8 to 425.8)	215.6 (190.6 to 245.0)	-46.4 (-53.5 to -38.0)
Peru	111 453 (95 721 to 129 931)	45 025 (37 198 to 53 625)	-59.6 (-68.0 to -49.5)	419.7 (366.9 to 481.2)	133.8 (111.1 to 159.5)	-68.1 (-74.5 to -60.4)
**Caribbean**	**186 163 (158 628 to 214 222)**	**108 417 (94 474 to 124 636)**	**-41.8 (-50.8 to -30.5)**	**482.6 (414.2 to 553.3)**	**249.0 (215.2 to 287.1)**	**-48.4 (-56.5 to -38.3)**
Antigua and Barbuda	230 (210 to 253)	233 (206 to 263)	1.4 (-13.1 to 18.8)	372.7 (340.1 to 408.1)	282.3 (244.3 to 323.1)	-24.3 (-35.9 to -10.1)
The Bahamas	1 305 (1 192 to 1 425)	1 347 (1 200 to 1 511)	3.2 (-10.3 to 18.2)	482.4 (442.4 to 523.7)	359.4 (320.5 to 403.7)	-25.5 (-35.2 to -15.0)
Barbados	806 (740 to 875)	510 (453 to 573)	-36.7 (-46.0 to -27.0)	312.9 (288.4 to 340.4)	187.5 (165.6 to 212.5)	-40.1 (-48.9 to -30.6)
Belize	1 158 (1 037 to 1 305)	1 485 (1 345 to 1 639)	28.3 (9.5 to 47.9)	494.5 (448.3 to 550.3)	356.4 (323.5 to 393.0)	-27.9 (-37.2 to -17.2)
Bermuda	219 (201 to 239)	83 (74 to 93)	-62.0 (-66.8 to -56.4)	395.1 (358.5 to 435.5)	147.4 (129.6 to 167.7)	-62.7 (-67.9 to -56.6)
Cuba	30 798 (29 146 to 32 605)	12 981 (11 404 to 14 792)	-57.8 (-63.4 to -51.7)	279.3 (264.4 to 297.0)	126.3 (111.1 to 142.7)	-54.8 (-60.7 to -48.4)
Dominica	281 (257 to 306)	231 (205 to 260)	-17.9 (-28.8 to -5.0)	366.2 (336.8 to 398.0)	361.3 (317.3 to 413.6)	-1.3 (-14.8 to 15.2)
Dominican Republic	22 552 (19 725 to 25 632)	15 632 (13 180 to 18 576)	-30.7 (-43.9 to -15.1)	264.8 (234.6 to 297.1)	149.3 (126.3 to 177.2)	-43.6 (-54.1 to -31.5)
Grenada	490 (445 to 547)	347 (315 to 384)	-29.1 (-39.3 to -18.9)	554.1 (507.5 to 609.6)	325.5 (293.1 to 362.8)	-41.3 (-49.6 to -32.2)
Guyana	3 777 (3 485 to 4 077)	3 010 (2 582 to 3 434)	-20.3 (-33.1 to -7.4)	452.1 (420.8 to 484.5)	393.9 (337.9 to 447.7)	-12.9 (-26.2 to 1.2)
Haiti	101 730 (75 705 to 129 076)	59 993 (47 134 to 75 273)	-41.0 (-55.7 to -18.2)	1 204.6 (918.3 to 1 483.7)	458.3 (364.2 to 571.2)	-62.0 (-70.8 to -49.0)
Jamaica	1 750 (1 545 to 1 971)	1 609 (1 334 to 1 977)	-8.1 (-27.1 to 15.9)	68.5 (61.1 to 76.4)	56.8 (47.4 to 69.0)	-17.1 (-33.6 to 2.7)
Puerto Rico	6 752 (6 334 to 7 158)	1 939 (1 740 to 2 148)	-71.3 (-74.5 to -67.4)	186.6 (175.1 to 198.2)	57.3 (51.2 to 63.7)	-69.3 (-73.0 to -65.0)
Saint Lucia	628 (574 to 688)	485 (437 to 536)	-22.7 (-32.8 to -11.3)	438.4 (403.6 to 476.3)	274.8 (245.9 to 305.3)	-37.3 (-45.2 to -28.2)
Saint Vincent and the Grenadines	472 (425 to 522)	385 (347 to 424)	-18.6 (-29.4 to -5.8)	407.2 (369.3 to 445.2)	344.0 (307.8 to 381.3)	-15.5 (-26.8 to -2.5)
Suriname	1 907 (1 712 to 2 122)	1 626 (1 422 to 1 857)	-14.8 (-28.0 to 0.6)	452.4 (407.7 to 502.0)	288.1 (252.4 to 328.9)	-36.3 (-45.9 to -25.1)
Trinidad and Tobago	4 600 (4 308 to 4 941)	2 418 (1 921 to 2 970)	-47.4 (-58.3 to -34.6)	360.8 (338.9 to 385.4)	183.8 (147.2 to 223.7)	-49.1 (-59.5 to -37.1)
Virgin Islands	336 (299 to 376)	203 (178 to 233)	-39.8 (-48.1 to -29.5)	311.8 (277.1 to 347.8)	194.3 (170.6 to 223.6)	-37.7 (-46.5 to -26.6)
**Central Latin America**	**661 167 (646 591 to 675 463)**	**377 268 (361 859 to 394 311)**	**-42.9 (-45.7 to -40.1)**	**361.0 (354.1 to 368.2)**	**146.1 (140.1 to 152.8)**	**-59.5 (-61.5 to -57.6)**
Colombia	133 010 (124 815 to 141 571)	66 925 (57 544 to 77 133)	-49.7 (-57.3 to -40.8)	359.0 (338.9 to 380.2)	138.1 (118.0 to 159.5)	-61.5 (-67.5 to -54.7)
Costa Rica	10 868 (10 204 to 11 630)	7 717 (6 938 to 8 649)	-29.0 (-37.5 to -18.8)	333.9 (315.6 to 354.8)	163.7 (147.3 to 183.1)	-51.0 (-56.6 to -44.2)
El Salvador	16 018 (14 251 to 18 279)	13 803 (11 052 to 17 695)	-13.8 (-33.6 to 13.0)	274.2 (245.6 to 308.3)	215.5 (173.8 to 274.5)	-21.4 (-38.7 to 2.9)
Guatemala	28 960 (27 086 to 30 909)	41 536 (36 421 to 47 285)	43.4 (24.3 to 67.3)	370.3 (349.5 to 391.6)	240.9 (212.1 to 272.8)	-34.9 (-43.2 to -25.0)
Honduras	25 288 (21 025 to 29 281)	16 195 (12 720 to 20 254)	-36.0 (-51.1 to -11.3)	458.0 (377.3 to 527.9)	167.0 (132.3 to 208.0)	-63.5 (-72.2 to -50.1)
Mexico	346 851 (336 897 to 356 814)	173 325 (168 260 to 179 042)	-50.0 (-52.0 to -47.7)	368.5 (359.2 to 377.7)	136.1 (132.1 to 140.7)	-63.1 (-64.4 to -61.5)
Nicaragua	16 496 (14 486 to 18 762)	8 725 (7 469 to 10 425)	-47.1 (-56.0 to -35.4)	360.8 (317.8 to 408.2)	131.3 (112.9 to 157.0)	-63.6 (-69.4 to -55.8)
Panama	11 150 (10 288 to 12 058)	7 366 (6 505 to 8 304)	-33.9 (-42.4 to -24.1)	433.5 (401.7 to 465.6)	189.2 (166.5 to 214.3)	-56.4 (-61.9 to -49.7)
Venezuela	72 526 (68 273 to 77 125)	41 675 (34 999 to 49 104)	-42.5 (-52.1 to -31.3)	339.9 (321.6 to 359.8)	135.1 (113.6 to 159.1)	-60.3 (-66.9 to -52.7)
**Tropical Latin America**	**679 939 (630 002 to 712 600)**	**355 832 (343 171 to 366 634)**	**-47.7 (-50.4 to -44.3)**	**397.8 (371.0 to 416.3)**	**166.8 (160.5 to 172.2)**	**-58.1 (-60.2 to -55.6)**
Brazil	671 224 (621 493 to 703 658)	346 520 (333 865 to 357 082)	-48.4 (-51.1 to -45.0)	403.8 (376.1 to 422.5)	168.5 (161.8 to 173.9)	-58.3 (-60.4 to -55.7)
Paraguay	8 715 (7 597 to 9 816)	9 312 (7 359 to 11 559)	6.8 (-17.4 to 37.6)	189.2 (166.6 to 211.8)	128.7 (102.3 to 159.3)	-32.0 (-46.9 to -12.8)
**North Africa and Middle East**	**1 350 036 (1 060 126 to 1 583 041)**	**737 672 (676 801 to 805 010)**	**-45.4 (-54.6 to -29.7)**	**302.2 (239.3 to 352.5)**	**117.2 (107.6 to 127.7)**	**-61.2 (-67.6 to -50.5)**
**North Africa and Middle East**	**1 350 036 (1 060 126 to 1 583 041)**	**737 672 (676 801 to 805 010)**	**-45.4 (-54.6 to -29.7)**	**302.2 (239.3 to 352.5)**	**117.2 (107.6 to 127.7)**	**-61.2 (-67.6 to -50.5)**
Afghanistan	116 688 (32 813 to 179 367)	147 099 (114 208 to 187 151)	26.1 (-22.0 to 357.6)	848.2 (234.8 to 1 259.5)	332.2 (259.9 to 414.1)	-60.8 (-75.1 to 46.6)
Algeria	69 431 (53 768 to 84 057)	32 392 (25 687 to 38 655)	-53.3 (-63.9 to -39.6)	209.0 (164.3 to 251.2)	79.3 (62.7 to 94.1)	-62.1 (-70.0 to -51.5)
Bahrain	599 (520 to 680)	521 (453 to 602)	-13.0 (-28.9 to 6.4)	112.9 (99.3 to 126.9)	38.4 (33.1 to 44.3)	-66.0 (-71.9 to -59.0)
Egypt	133 615 (108 617 to 158 314)	99 274 (82 119 to 119 833)	-25.7 (-42.4 to -1.8)	195.7 (159.9 to 230.0)	94.7 (78.7 to 113.6)	-51.6 (-62.0 to -37.0)
Iran	231 673 (201 883 to 256 054)	62 772 (60 258 to 66 995)	-72.9 (-75.8 to -68.4)	296.8 (258.4 to 328.5)	80.5 (77.1 to 86.2)	-72.9 (-75.8 to -68.3)

Location	All-age years of life lost (95% UI)			Age-standardised years of life lost rates per 100,000 (95% UI)		
	1990	2017	Percentage change between 1990 and 2017	1990	2017	Percentage change between 1990 and 2017
Iraq	50 251 (34 111 to 61 401)	41 600 (35 585 to 49 572)	-17.2 (-36.5 to 19.0)	218.5 (152.7 to 264.1)	79.3 (68.4 to 93.3)	-63.7 (-71.6 to -49.4)
Jordan	9 251 (7 662 to 10 915)	7 513 (6 157 to 9 232)	-18.8 (-37.7 to 9.6)	183.4 (154.0 to 214.8)	63.8 (52.5 to 78.2)	-65.2 (-73.2 to -53.5)
Kuwait	2 133 (1 855 to 2 467)	1 340 (1 186 to 1 509)	-37.2 (-46.0 to -27.4)	108.7 (94.9 to 126.1)	32.9 (29.1 to 37.2)	-69.7 (-74.2 to -65.0)
Lebanon	6 831 (5 478 to 8 361)	5 203 (4 110 to 6 425)	-23.8 (-43.9 to 2.3)	140.8 (114.9 to 171.8)	57.1 (45.4 to 70.2)	-59.5 (-70.0 to -46.1)
Libya	9 810 (7 770 to 12 007)	7 458 (5 685 to 10 000)	-24.0 (-45.0 to 10.1)	189.3 (151.3 to 227.9)	104.3 (79.7 to 138.9)	-44.9 (-59.9 to -21.3)
Morocco	93 251 (71 831 to 114 083)	38 414 (29 839 to 48 031)	-58.8 (-69.0 to -45.4)	298.0 (230.9 to 363.2)	109.8 (85.1 to 137.2)	-63.2 (-72.2 to -51.3)
Palestine	4 786 (3 707 to 6 033)	2 599 (2 119 to 3 525)	-45.7 (-59.2 to -24.9)	165.2 (131.5 to 201.4)	47.0 (39.0 to 63.1)	-71.5 (-78.0 to -61.6)
Oman	8 292 (6 385 to 10 461)	5 893 (4 829 to 7 256)	-28.9 (-48.8 to -1.5)	342.7 (270.6 to 427.2)	137.3 (113.7 to 168.9)	-59.9 (-71.0 to -45.3)
Qatar	701 (574 to 851)	2 012 (1 604 to 2 520)	186.8 (114.6 to 291.2)	154.5 (127.9 to 186.1)	77.7 (63.3 to 94.8)	-49.7 (-61.9 to -33.4)
Saudi Arabia	89 331 (65 315 to 119 021)	69 172 (52 377 to 93 210)	-22.6 (-50.0 to 25.6)	450.7 (338.3 to 582.2)	183.2 (143.2 to 237.0)	-59.4 (-73.3 to -36.0)
Sudan	201 284 (119 368 to 292 111)	89 756 (67 919 to 118 582)	-55.4 (-71.0 to -25.2)	647.1 (391.1 to 921.4)	171.6 (131.2 to 221.8)	-73.5 (-82.4 to -57.0)
Syria	23 853 (19 020 to 29 774)	9 199 (7 329 to 11 399)	-61.4 (-72.4 to -45.9)	143.2 (116.6 to 173.6)	46.5 (37.2 to 57.4)	-67.5 (-76.1 to -55.9)
Tunisia	19 204 (15 322 to 24 112)	5 289 (3 976 to 6 700)	-72.5 (-80.4 to -62.3)	187.0 (150.2 to 232.6)	49.5 (37.1 to 63.1)	-73.5 (-81.2 to -63.9)
Turkey	180 924 (142 014 to 225 198)	49 143 (42 608 to 56 695)	-72.8 (-79.1 to -63.7)	266.7 (210.1 to 330.4)	65.2 (56.3 to 75.6)	-75.5 (-81.2 to -67.4)
United Arab Emirates	3 730 (2 803 to 5 009)	7 936 (6 167 to 10 159)	112.7 (41.2 to 220.9)	193.8 (146.2 to 258.0)	104.5 (82.0 to 133.5)	-46.1 (-63.4 to -19.8)
Yemen	93 528 (38 552 to 139 377)	52 397 (38 897 to 70 789)	-44.0 (-64.0 to 38.2)	436.0 (176.8 to 645.2)	140.5 (104.9 to 190.1)	-67.8 (-78.5 to -18.4)
**South Asia**	**11 565 562 (9 427 496 to 13 346 075)**	**5 273 794 (4 819 285 to 5 744 698)**	**-54.4 (-60.7 to -43.7)**	**817.6 (676.9 to 935.0)**	**295.1 (270.1 to 321.2)**	**-63.9 (-68.8 to -56.2)**
**South Asia**	**11 565 562 (9 427 496 to 13 346 075)**	**5 273 794 (4 819 285 to 5 744 698)**	**-54.4 (-60.7 to -43.7)**	**817.6 (676.9 to 935.0)**	**295.1 (270.1 to 321.2)**	**-63.9 (-68.8 to -56.2)**
Bangladesh	3 931 285 (2 866 019 to 4 919 180)	943 569 (725 416 to 1 199 634)	-76.0 (-81.9 to -65.5)	2 348.5 (1 752.6 to 2 896.8)	613.5 (470.3 to 780.8)	-73.9 (-80.3 to -62.9)
Bhutan	3 587 (2 621 to 4 677)	1 332 (961 to 1 681)	-62.9 (-75.0 to -47.3)	489.4 (366.1 to 622.7)	138.1 (102.8 to 174.0)	-71.8 (-80.3 to -60.9)
India	6 253 458 (5 218 182 to 7 171 120)	3 349 439 (3 093 096 to 3 649 785)	-46.4 (-53.7 to -36.0)	598.5 (508.6 to 680.0)	245.8 (226.9 to 268.1)	-58.9 (-64.2 to -51.4)
Nepal	212 147 (140 575 to 290 466)	53 523 (39 116 to 67 787)	-74.8 (-83.5 to -57.8)	773.2 (528.7 to 1 037.6)	174.2 (127.3 to 218.9)	-77.5 (-85.0 to -63.8)
Pakistan	1 165 085 (781 300 to 1 594 722)	925 931 (718 042 to 1 149 911)	-20.5 (-44.0 to 18.4)	731.8 (503.7 to 983.5)	367.9 (288.2 to 453.5)	-49.7 (-63.9 to -27.7)
**Southeast Asia, East Asia, and Oceania**	**16 954 245 (15 583 135 to 18 123 505)**	**5 093 359 (4 890 553 to 5 311 978)**	**-70.0 (-71.9 to -67.6)**	**915.7 (843.6 to 978.0)**	**289.0 (276.4 to 302.7)**	**-68.4 (-70.5 to -66.0)**
**East Asia**	**12 975 150 (12 047 581 to 13 885 072)**	**3 244 972 (3 087 127 to 3 385 405)**	**-75.0 (-76.7 to -73.0)**	**1 022.2 (949.6 to 1 094.6)**	**297.3 (283.2 to 311.9)**	**-70.9 (-72.9 to -68.7)**
China	12 565 842 (11 661 117 to 13 465 630)	3 079 099 (2 935 184 to 3 218 715)	-75.5 (-77.2 to -73.5)	1 040.0 (965.6 to 1 114.8)	297.2 (282.7 to 311.9)	-71.4 (-73.4 to -69.2)
North Korea	94 603 (76 380 to 116 575)	91 373 (72 784 to 111 664)	-3.4 (-26.1 to 28.4)	461.9 (373.3 to 568.1)	446.5 (352.9 to 547.3)	-3.3 (-25.0 to 27.2)
Taiwan (Province of China)	98 536 (94 505 to 102 818)	22 225 (20 699 to 23 973)	-77.4 (-79.2 to -75.5)	487.4 (465.7 to 510.4)	95.2 (88.3 to 103.3)	-80.5 (-82.1 to -78.6)
**Oceania**	**66 329 (54 475 to 78 918)**	**114 557 (93 052 to 138 198)**	**72.7 (40.1 to 113.0)**	**917.8 (759.8 to 1 088.7)**	**828.2 (672.7 to 997.9)**	**-9.8 (-26.6 to 10.6)**
American Samoa	254 (217 to 294)	206 (179 to 237)	-18.9 (-33.7 to -1.3)	488.6 (423.1 to 555.4)	362.7 (317.2 to 415.5)	-25.8 (-38.2 to -10.4)
Federated States of Micronesia	635 (521 to 771)	483 (309 to 635)	-23.9 (-50.2 to 3.5)	579.8 (477.2 to 710.9)	448.2 (278.6 to 592.5)	-22.7 (-50.5 to 5.1)
Fiji	3 552 (3 017 to 4 166)	3 798 (3 215 to 4 524)	6.9 (-14.6 to 34.7)	426.7 (367.4 to 495.2)	410.1 (347.5 to 488.5)	-3.9 (-22.7 to 20.6)
Guam	435 (381 to 492)	419 (369 to 470)	-3.6 (-18.7 to 14.6)	296.7 (261.7 to 333.3)	252.1 (221.5 to 282.8)	-15.0 (-28.2 to 0.8)
Kiribati	373 (299 to 455)	377 (300 to 465)	1.0 (-22.4 to 34.3)	424.3 (350.3 to 501.4)	294.0 (236.2 to 360.3)	-30.7 (-45.8 to -9.6)
Marshall Islands	297 (250 to 348)	340 (277 to 417)	14.8 (-10.1 to 43.5)	648.5 (557.3 to 750.8)	580.8 (475.2 to 708.8)	-10.4 (-29.4 to 12.1)
Northern Mariana Islands	198 (165 to 236)	150 (129 to 173)	-24.3 (-39.9 to -4.3)	399.0 (333.5 to 474.1)	343.0 (294.8 to 393.0)	-14.0 (-30.6 to 7.1)
Papua New Guinea	51 776 (41 477 to 63 416)	95 946 (75 806 to 117 639)	85.3 (46.1 to 135.5)	1 128.9 (905.9 to 1 370.4)	936.9 (738.1 to 1 150.9)	-17.0 (-34.5 to 4.7)
Samoa	589 (483 to 716)	526 (410 to 659)	-10.7 (-31.8 to 14.8)	335.4 (276.5 to 406.7)	254.1 (197.2 to 317.8)	-24.3 (-42.9 to -2.1)
Solomon Islands	2 511 (1 992 to 3 119)	3 771 (3 002 to 4 593)	50.2 (15.5 to 91.0)	701.3 (561.0 to 877.1)	559.6 (444.1 to 685.5)	-20.2 (-38.9 to 2.8)
Tonga	368 (303 to 440)	392 (324 to 468)	6.5 (-16.3 to 39.1)	349.7 (292.6 to 411.2)	371.1 (305.4 to 443.4)	6.1 (-16.3 to 37.6)
Vanuatu	964 (717 to 1 246)	1 836 (1 308 to 2 503)	90.5 (35.9 to 166.4)	625.1 (456.1 to 807.2)	606.5 (435.4 to 829.8)	-3.0 (-29.9 to 34.5)
**Southeast Asia**	**3 912 766 (3 370 720 to 4 386 229)**	**1 733 830 (1 631 541 to 1 853 116)**	**-55.7 (-60.7 to -48.6)**	**706.3 (612.4 to 786.3)**	**277.1 (260.1 to 297.1)**	**-60.8 (-65.0 to -54.9)**
Cambodia	236 180 (186 966 to 284 517)	81 456 (68 108 to 97 900)	-65.5 (-73.3 to -53.9)	1 523.2 (1 238.7 to 1 797.1)	483.1 (407.0 to 574.9)	-68.3 (-74.9 to -58.8)
Indonesia	1 339 494 (1 129 871 to 1 505 797)	362 304 (330 169 to 396 522)	-73.0 (-76.0 to -68.8)	602.3 (511.5 to 674.8)	153.4 (139.0 to 168.7)	-74.5 (-77.3 to -71.0)
Laos	63 411 (45 662 to 81 622)	32 308 (24 541 to 40 596)	-49.1 (-63.5 to -32.1)	1 169.0 (882.8 to 1 476.7)	427.6 (327.3 to 535.2)	-63.4 (-73.4 to -52.7)
Malaysia	44 673 (38 048 to 51 336)	43 924 (38 462 to 50 627)	-1.7 (-19.6 to 23.2)	234.2 (204.4 to 266.2)	140.5 (123.8 to 160.9)	-40.0 (-50.1 to -26.2)
Maldives	2 602 (1 909 to 3 303)	714 (593 to 817)	-72.6 (-78.9 to -62.7)	914.1 (697.9 to 1 119.2)	169.0 (142.4 to 193.3)	-81.5 (-85.2 to -76.3)
Mauritius	2 655 (2 483 to 2 851)	2 069 (1 863 to 2 295)	-22.1 (-30.9 to -11.9)	235.3 (220.5 to 251.2)	156.8 (141.1 to 175.0)	-33.4 (-41.1 to -24.6)
Myanmar	583 038 (432 069 to 755 036)	208 255 (169 552 to 250 879)	-64.3 (-74.2 to -50.2)	1 189.2 (892.3 to 1 528.8)	424.8 (342.7 to 515.5)	-64.3 (-74.2 to -50.5)
Philippines	349 397 (308 188 to 391 700)	329 143 (288 053 to 381 192)	-5.8 (-21.0 to 14.8)	451.8 (404.7 to 499.9)	300.7 (264.3 to 346.4)	-33.4 (-43.5 to -20.1)
Sri Lanka	66 278 (58 759 to 73 912)	46 240 (37 870 to 55 600)	-30.2 (-43.9 to -13.2)	381.8 (341.6 to 421.0)	213.7 (174.9 to 257.1)	-44.0 (-54.9 to -30.5)
Seychelles	522 (474 to 569)	578 (525 to 638)	10.9 (-2.5 to 26.8)	733.5 (669.5 to 796.8)	568.5 (512.4 to 628.7)	-22.5 (-31.9 to -11.5)
Thailand	409 439 (340 819 to 474 718)	238 696 (211 942 to 266 119)	-41.7 (-51.6 to -28.4)	690.8 (575.1 to 802.6)	381.7 (336.8 to 425.2)	-44.7 (-53.9 to -33.2)
Timor-Leste	9 230 (6 240 to 12 350)	4 728 (2 501 to 6 022)	-48.8 (-72.9 to -22.9)	843.0 (602.2 to 1 089.2)	310.3 (168.0 to 390.1)	-63.2 (-79.9 to -47.2)
Vietnam	800 653 (633 558 to 977 416)	381 135 (324 097 to 441 882)	-52.4 (-62.1 to -37.7)	970.0 (778.6 to 1 170.8)	433.2 (367.2 to 508.1)	-55.3 (-64.3 to -42.0)
**Sub-Saharan Africa**	**3 268 193 (2 515 457 to 3 971 977)**	**2 998 157 (2 627 149 to 3 384 240)**	**-8.3 (-25.6 to 21.6)**	**456.1 (363.2 to 545.6)**	**226.8 (202.3 to 252.6)**	**-50.3 (-58.8 to -36.1)**
**Central sub-Saharan Africa**	**720 057 (470 329 to 969 440)**	**661 771 (512 219 to 850 115)**	**-8.1 (-33.5 to 37.2)**	**831.4 (561.4 to 1 098.2)**	**399.0 (319.2 to 502.4)**	**-52.0 (-64.7 to -30.2)**
Angola	220 539 (115 320 to 331 147)	150 799 (111 708 to 198 679)	-31.6 (-57.0 to 23.5)	1 347.8 (734.5 to 1 985.1)	381.3 (292.5 to 481.9)	-71.7 (-81.4 to -50.1)
Central African Republic	32 250 (16 596 to 46 310)	36 631 (24 841 to 51 528)	13.6 (-25.4 to 127.2)	818.5 (443.7 to 1 140.0)	660.2 (458.3 to 913.9)	-19.3 (-45.0 to 55.8)
Congo (Brazzaville)	25 336 (17 824 to 34 648)	17 156 (12 492 to 22 775)	-32.3 (-53.0 to -0.1)	750.4 (550.8 to 995.8)	304.0 (224.3 to 397.1)	-59.5 (-70.8 to -42.9)
DR Congo	428 434 (273 815 to 606 769)	448 976 (315 715 to 625 124)	4.8 (-33.0 to 70.9)	703.2 (459.2 to 971.0)	402.5 (293.6 to 546.5)	-42.8 (-61.7 to -11.1)
Equatorial Guinea	6 688 (4 483 to 9 236)	3 565 (2 292 to 5 433)	-46.7 (-67.8 to -14.1)	1 006.9 (706.2 to 1 348.8)	217.8 (141.3 to 326.5)	-78.4 (-86.5 to -66.0)

Location	All-age years of life lost (95% UI)			Age-standardised years of life lost rates per 100,000 (95% UI)		
	1990	2017	Percentage change between 1990 and 2017	1990	2017	Percentage change between 1990 and 2017
Gabon	6 810 (4 819 to 9 396)	4 644 (3 484 to 6 063)	-31.8 (-52.7 to 0.7)	539.4 (396.9 to 726.5)	251.9 (191.4 to 324.3)	-53.3 (-66.7 to -32.8)
**Eastern sub-Saharan Africa**	**1 196 303 (859 184 to 1 499 773)**	**872 973 (782 262 to 985 073)**	**-27.0 (-41.6 to 2.8)**	**422.5 (310.4 to 520.2)**	**179.0 (162.6 to 198.8)**	**-57.6 (-65.6 to -42.5)**
Burundi	37 396 (26 832 to 48 856)	31 566 (23 845 to 41 395)	-15.6 (-40.8 to 23.3)	475.8 (366.2 to 597.6)	225.2 (177.7 to 285.6)	-52.7 (-64.8 to -35.0)
Comoros	2 247 (1 738 to 2 809)	1 069 (859 to 1 356)	-52.4 (-64.9 to -35.9)	363.0 (287.3 to 440.1)	140.8 (114.9 to 178.7)	-61.2 (-70.4 to -49.0)
Djibouti	2 468 (1 402 to 3 374)	1 783 (1 313 to 2 559)	-27.7 (-51.6 to 26.4)	362.9 (213.8 to 481.3)	151.1 (113.0 to 212.0)	-58.4 (-71.5 to -29.8)
Eritrea	19 953 (9 334 to 29 458)	16 445 (12 618 to 21 091)	-17.6 (-47.3 to 78.9)	496.6 (251.8 to 694.4)	245.5 (192.0 to 308.5)	-50.6 (-66.9 to -2.3)
Ethiopia	417 751 (272 116 to 549 854)	209 033 (175 332 to 252 140)	-50.0 (-63.5 to -19.0)	546.4 (373.1 to 701.1)	161.5 (139.4 to 189.1)	-70.4 (-77.9 to -55.2)
Kenya	68 003 (54 225 to 80 739)	67 631 (61 120 to 76 014)	-0.5 (-16.5 to 24.7)	218.1 (175.6 to 256.3)	131.4 (120.1 to 145.2)	-39.7 (-48.7 to -26.3)
Madagascar	75 133 (59 113 to 92 644)	57 674 (43 606 to 76 104)	-23.2 (-44.0 to 8.5)	442.3 (356.4 to 534.1)	178.6 (140.0 to 229.1)	-59.6 (-69.3 to -45.7)
Malawi	72 342 (25 549 to 110 008)	39 483 (30 700 to 51 032)	-45.4 (-66.1 to 54.9)	473.9 (169.6 to 708.4)	185.2 (148.5 to 231.1)	-60.9 (-74.6 to 9.0)
Mozambique	114 063 (80 369 to 151 786)	78 177 (62 291 to 96 979)	-31.5 (-52.0 to 0.4)	542.1 (395.7 to 701.8)	222.4 (183.1 to 268.0)	-59.0 (-70.0 to -42.4)
Rwanda	47 463 (34 652 to 61 100)	24 210 (18 951 to 31 580)	-49.0 (-64.1 to -20.4)	457.6 (353.0 to 565.3)	160.1 (129.1 to 202.3)	-65.0 (-73.9 to -50.1)
Somalia	50 309 (14 485 to 85 216)	51 411 (34 448 to 74 815)	2.2 (-44.3 to 247.3)	468.2 (147.7 to 770.9)	236.8 (163.6 to 335.3)	-49.4 (-71.3 to 56.3)
South Sudan	33 542 (11 972 to 58 290)	42 791 (29 454 to 59 090)	27.6 (-22.1 to 205.8)	417.2 (158.5 to 704.3)	310.8 (220.5 to 416.8)	-25.5 (-52.7 to 66.0)
Tanzania	99 563 (45 615 to 138 312)	129 267 (98 497 to 169 893)	29.8 (-14.2 to 197.3)	265.1 (128.4 to 358.7)	181.0 (144.0 to 228.4)	-31.7 (-52.8 to 46.7)
Uganda	91 771 (61 363 to 128 874)	77 517 (59 728 to 99 055)	-15.5 (-42.1 to 30.5)	318.9 (221.5 to 432.1)	150.2 (120.7 to 188.1)	-52.9 (-65.6 to -31.1)
Zambia	63 692 (36 714 to 88 808)	44 369 (34 229 to 57 908)	-30.3 (-54.6 to 27.1)	528.7 (315.4 to 714.4)	201.3 (161.0 to 252.7)	-61.9 (-73.6 to -32.6)
**Southern sub-Saharan Africa**	**158 768 (142 781 to 174 303)**	**136 892 (126 207 to 147 965)**	**-13.8 (-23.7 to -1.2)**	**282.4 (256.4 to 309.6)**	**172.1 (159.3 to 185.8)**	**-39.0 (-45.9 to -30.8)**
Botswana	3 004 (2 335 to 3 700)	2 564 (2 131 to 3 087)	-14.7 (-34.6 to 12.5)	221.5 (173.8 to 272.7)	111.3 (93.2 to 133.5)	-49.8 (-60.9 to -34.3)
Lesotho	5 029 (4 062 to 6 117)	6 364 (5 128 to 7 829)	26.6 (-2.0 to 67.2)	271.3 (222.4 to 323.6)	319.3 (258.8 to 387.5)	17.7 (-8.8 to 52.6)
Namibia	4 167 (3 306 to 5 012)	3 919 (3 066 to 4 978)	-6.0 (-28.9 to 24.7)	280.2 (223.8 to 331.5)	161.7 (127.9 to 202.8)	-42.3 (-55.3 to -24.6)
South Africa	112 959 (102 346 to 124 351)	76 089 (69 938 to 83 131)	-32.6 (-40.2 to -24.0)	290.8 (264.8 to 319.2)	134.7 (124.3 to 147.0)	-53.7 (-58.5 to -48.3)
Swaziland	2 623 (2 082 to 3 223)	3 211 (2 492 to 4 074)	22.4 (-10.8 to 71.9)	298.8 (245.0 to 355.8)	278.3 (218.0 to 350.0)	-6.8 (-31.5 to 26.4)
Zimbabwe	30 986 (24 582 to 36 868)	44 747 (36 773 to 53 663)	44.4 (10.5 to 89.4)	257.5 (214.8 to 298.0)	279.7 (235.3 to 332.5)	8.6 (-13.9 to 37.9)
**Western sub-Saharan Africa**	**1 193 065 (935 134 to 1 445 592)**	**1 326 520 (1 104 823 to 1 612 956)**	**11.2 (-12.6 to 50.6)**	**413.7 (333.8 to 493.8)**	**224.6 (190.0 to 266.6)**	**-45.7 (-56.1 to -28.8)**
Benin	33 602 (24 230 to 43 451)	29 761 (21 659 to 41 298)	-11.4 (-40.1 to 29.9)	438.0 (333.5 to 555.2)	199.3 (151.5 to 262.3)	-54.5 (-66.9 to -37.7)
Burkina Faso	51 585 (38 233 to 66 799)	90 728 (64 050 to 124 761)	75.9 (18.9 to 167.4)	354.2 (272.1 to 442.3)	300.0 (224.8 to 395.2)	-15.3 (-39.9 to 22.6)
Cameroon	48 533 (35 757 to 64 552)	59 466 (43 577 to 77 985)	22.5 (-14.9 to 83.7)	335.3 (262.2 to 425.5)	181.8 (139.0 to 230.1)	-45.8 (-60.3 to -23.3)
Cape Verde	1 097 (892 to 1 333)	1 479 (1 247 to 1 782)	34.9 (4.8 to 73.3)	273.7 (233.2 to 320.5)	273.6 (230.6 to 331.2)	-0.1 (-19.8 to 26.0)
Chad	38 740 (25 890 to 54 157)	74 414 (54 933 to 101 546)	92.1 (30.4 to 192.9)	402.6 (280.3 to 548.0)	316.1 (241.9 to 412.9)	-21.5 (-44.1 to 11.6)
Cote d'Ivoire	66 054 (48 247 to 86 876)	73 634 (56 781 to 95 330)	11.5 (-20.7 to 60.2)	371.4 (285.1 to 471.4)	238.4 (191.3 to 297.0)	-35.8 (-52.6 to -11.8)
The Gambia	5 386 (3 768 to 7 268)	4 463 (3 049 to 6 186)	-17.1 (-44.4 to 19.7)	364.1 (265.3 to 480.2)	179.0 (129.6 to 237.9)	-50.8 (-65.0 to -31.9)
Ghana	38 702 (30 617 to 49 810)	47 394 (37 603 to 58 922)	22.5 (-10.7 to 69.4)	207.1 (170.1 to 255.5)	148.0 (119.9 to 180.7)	-28.5 (-45.1 to -5.6)
Guinea	62 558 (41 261 to 87 223)	41 972 (31 847 to 54 479)	-32.9 (-55.5 to 5.2)	628.7 (435.7 to 857.5)	270.8 (212.3 to 339.4)	-56.9 (-70.3 to -35.2)
Guinea-Bissau	9 736 (6 389 to 14 424)	6 266 (4 308 to 8 741)	-35.6 (-56.6 to -1.8)	660.3 (450.8 to 944.4)	277.9 (200.0 to 377.6)	-57.9 (-69.4 to -39.0)
Liberia	17 323 (12 070 to 23 038)	8 925 (6 073 to 13 161)	-48.5 (-65.8 to -16.6)	565.1 (408.1 to 736.4)	160.2 (116.9 to 225.2)	-71.6 (-80.1 to -56.5)
Mali	92 766 (64 179 to 132 346)	120 114 (83 610 to 170 562)	29.5 (-14.3 to 103.9)	651.1 (466.1 to 901.4)	376.3 (271.6 to 522.9)	-42.2 (-60.1 to -13.2)
Mauritania	6 514 (4 977 to 8 311)	5 493 (4 103 to 7 764)	-15.7 (-41.9 to 25.6)	246.5 (199.1 to 303.6)	119.8 (92.6 to 161.3)	-51.4 (-64.2 to -31.9)
Niger	110 835 (71 734 to 160 123)	92 983 (64 038 to 131 550)	-16.1 (-47.2 to 35.9)	790.3 (528.5 to 1 113.6)	272.4 (199.5 to 369.0)	-65.5 (-76.8 to -47.0)
Nigeria	501 996 (369 830 to 659 269)	596 655 (426 343 to 815 534)	18.9 (-19.7 to 80.5)	383.4 (290.7 to 498.0)	202.8 (149.8 to 273.2)	-47.1 (-63.0 to -22.9)
Sao Tome and Principe	437 (327 to 570)	217 (172 to 273)	-50.2 (-65.1 to -29.5)	255.8 (201.7 to 321.6)	105.0 (84.9 to 131.1)	-58.9 (-70.0 to -44.1)
Senegal	50 899 (36 270 to 69 906)	32 349 (22 721 to 45 512)	-36.4 (-55.8 to -1.5)	443.1 (327.1 to 587.1)	186.7 (135.6 to 251.6)	-57.9 (-69.2 to -39.0)
Sierra Leone	37 490 (26 822 to 51 210)	27 065 (19 639 to 35 863)	-27.8 (-52.1 to 10.8)	645.5 (475.6 to 870.2)	272.5 (204.2 to 356.2)	-57.8 (-70.8 to -38.2)
Togo	18 778 (13 944 to 24 351)	13 129 (9 760 to 17 815)	-30.1 (-51.3 to 2.4)	345.1 (268.7 to 434.4)	155.2 (117.0 to 204.3)	-55.0 (-67.1 to -37.2)
